# TFAP2A downregulation mediates tumor-suppressive effect of miR-8072 in triple-negative breast cancer via inhibiting* SNAI1* transcription

**DOI:** 10.1186/s13058-024-01858-x

**Published:** 2024-06-18

**Authors:** Yujie Fang, Yali Wang, Hongning Ma, Yuqi Guo, Rongrong Xu, Xixi Chen, Xuehua Chen, Ye Lv, Pu Li, Yujing Gao

**Affiliations:** 1 Key Laboratory of Fertility Preservation and Maintenance of Ministry of Education, Department of Biochemistry and Molecular Biology, School of Basic Medical Sciences, Ningxia Medical University, Yinchuan, China; 2 Oncology Department of Cancer Hospital, General Hospital, Ningxia Medical University, Yinchuan, China; 3 Department of Pediatrics, Ruijin Hospital, Shanghai Jiao Tong University School of Medicine, Shanghai, China; 4 Central Laboratory of People’s Hospital of Ningxia Hui Autonomous Region, Yinchuan, China; 5 National Health Commission Key Laboratory of Metabolic Cardiovascular Diseases Research, Ningxia Medical University, Yinchuan, China

**Keywords:** Triple-negative breast cancer, TFAP2A, SNAI1, miR-8072, Tumorigenesis

## Abstract

**Background:**

Triple-negative breast cancer (TNBC) represents a highly aggressive subset of breast malignancies characterized by its challenging clinical management and unfavorable prognosis. While TFAP2A, a member of the AP-2 transcription factor family, has been implicated in maintaining the basal phenotype of breast cancer, its precise regulatory role in TNBC remains undefined.

**Methods:**

In vitro assessments of TNBC cell growth and migratory potential were conducted using MTS, colony formation, and EdU assays. Quantitative PCR was employed to analyze mRNA expression levels, while Western blot was utilized to evaluate protein expression and phosphorylation status of AKT and ERK. The post-transcriptional regulation of TFAP2A by miR-8072 and the transcriptional activation of *SNAI1* by TFAP2A were investigated through luciferase reporter assays. A xenograft mouse model was employed to assess the in vivo growth capacity of TNBC cells.

**Results:**

Selective silencing of TFAP2A significantly impeded the proliferation and migration of TNBC cells, with elevated TFAP2A expression observed in breast cancer tissues. Notably, TNBC patients exhibiting heightened TFAP2A levels experienced abbreviated overall survival. Mechanistically, TFAP2A was identified as a transcriptional activator of *SNAI1*, a crucial regulator of epithelial-mesenchymal transition (EMT) and cellular proliferation, thereby augmenting the oncogenic properties of TFAP2A in TNBC. Moreover, miR-8072 was unveiled as a negative regulator of TFAP2A, exerting potent inhibitory effects on TNBC cell growth and migration. Importantly, the tumor-suppressive actions mediated by the miR-8072/TFAP2A axis were intricately associated with the attenuation of AKT/ERK signaling cascades and the blockade of EMT processes.

**Conclusions:**

Our findings unravel the role and underlying molecular mechanism of TFAP2A in driving tumorigenesis of TNBC. Targeting the TFAP2A/SNAI1 pathway and utilizing miR-8072 as a suppressor represent promising therapeutic strategies for treating TNBC.

**Supplementary Information:**

The online version contains supplementary material available at 10.1186/s13058-024-01858-x.

## Background

Breast cancer is the most frequently diagnosed cancer among women. It has surpassed lung cancer as the leading cancer in women since 2020 [1]. Triple-negative breast cancer (TNBC) is a subtype characterized by the absence of estrogen and progesterone receptors, the deletion of the human epidermal growth factor 2 receptor, and notable heterogeneity. Although TNBC represents a relatively low incidence subtype compared to others, it exhibits a high recurrence rate, metastatic potential, and invasiveness, making it the most lethal form of breast cancer in young women [2]. The median survival for patients with metastatic TNBC is a mere 1 year statistically. Treatment for TNBC typically involves chemotherapy in both early and advanced stages. However, less than 30% of patients with metastatic breast cancer survive beyond five years after diagnosis, and nearly all patients with metastatic TNBC eventually succumb to the disease [3]. Currently, the lack of targeted drug therapies has prompted an intense search for molecular targets to effectively treat TNBC.

Transcription factor AP-2α (TFAP2A) is a member of the AP-2 family of transcription factors that include five members—TFAP2A (AP-2α), TFAP2B (AP-2β), TFAP2C (AP-2γ), TFAP2D (AP-2δ), and TFAP2E (AP-2ε). In tumor models, both TFAP2A and TFAP2C are important to cell proliferation and migration [4–7]. In breast cancer, TFAP2C induces expression of ERα as well as other ERα-associated genes while represses expression of basal-associated gene [8–10], playing crucial role in maintaining the luminal phenotype of breast cancer. Although TFAP2A shares 83% similarity in structure with TFAP2C, it is reported TFAP2A lacks transcriptional activity at luminal gene promoters, attributed to SUMOylation of TFAP2A in luminal breast cancer cells which blocks its ability to induce the expression of luminal genes but is crucial to maintain the basal breast cancer subtype [9]. In ER-positive breast cancer, a few studies have shown that TFAP2A promotes tumor progression [11, 12]. However, the precise role and mechanism of TFAP2A in TNBC remains unclear.

Epithelial mesenchymal transition (EMT) plays an essential role in normal embryonic development and is closely associated with tumor invasion and metastasis [13, 14]. Previous studies have shown in breast tumors EMT likely occurs within the basal phenotype, and this proclivity may be related to the high aggressiveness and metastatic capacity of these tumors [9, 15–17]. Inducers of the EMT include several transcription factors, such as SNAI1, Slug, Zeb1/2, and Twist. Many studies indicates SNAI1 are highly expressed in TNBC and implicated in the development and progression of TNBC [18–21], letting it become a target for the treatment and prevention of TNBC [22]. As an important transcription factor in maintaining basal-like subtype breast cancer, whether and how TFAP2A modulates EMT in TNBC is poorly understood.

MicroRNA (miRNA), a small non-coding RNA that exists in eukaryotic cells and is a small endogenous regulatory molecule that regulates gene expression after transcription. Owing to its important function in regulating cell differentiation, development, and homeostasis, deregulation of miRNA is associated with an increasing number of human diseases, particularly cancer [23]. Notably, delivery of miRNA by nanoparticles becomes a potential therapeutic way for effective and personalized medicine for cancer treatment [24, 25]. Therefore, identifying miRNAs with effective tumor-suppressive effect could provide new choice for cancer treatment, which is more import for TNBC, since it has less therapeutic choice.

In our current study, we have identified miR-8072 as a promising candidate for suppressing tumorigenesis in TNBC. We conducted experiments that demonstrated the significant impact of miR-8072 on TNBC cell proliferation and migration, highlighting its potential as a therapeutic inhibitor. Furthermore, our findings indicate that the downregulation of TFAP2A plays a crucial role in mediating the tumor-suppressive effects of miR-8072 by inhibiting the EMT process and AKT/ERK signaling pathways. This effect can be partly attributed to the reduced transcriptional activation of *SNAI1* by TFAP2A, a critical regulator of the EMT process.

## Materials and methods

### Cell culture and transfection

Human triple-negative breast cancer cell lines MDA-MB-231 and BT-549 were cultured in RPMI-1640 Medium (Gibco, USA) with 10% fetal bovine serum (FBS) (Hyclone, USA). HEK293T cells were cultured in DMEM medium supplemented with 10% FBS. Cells were maintained at 37 °C, 5% CO_2_ in a humidified incubator.

miR-8072 mimics, inhibitors and the corresponding control oligos were purchased from RiboBio Biotechnology Co. Ltd. (Guangzhou, China). Lentivirus containing miR-8072 mimics or inhibitors were produced by GeneChem Biotechnology Company (Shanghai, China). siRNAs targeting TFAP2A were designed and synthesized by GenePharma Company (Shanghai, China). The sense sequences of the siRNAs are as follows: siNC (negative control), 5′-UUCUCCGAACGUGUCACGUTT-3′; siTFAP2A#1, 5′-GCAAGAUCCUUACUCCCACTT-3′; siTFAP2A#2, 5′-CCUGCUCACAUCACUAGUATT-3′; siTFAP2A#3, 5′-CCCAAUGAGCAAGUGACAATT-3′. Cell transfection was performed as described previously [26]. For viral infection, cells with 30–40% confluence were transduced with lentivirus aforementioned and selected with 2 μg/mL puromycin. After two weeks of screening, cells were maintained in RPMI 1640 complete medium containing 0.5 μg/mL puromycin.

### Antibodies

The AKT(#4691), p-AKT(Ser 473, #9271), ERK1/2(#4695), p-ERK1/2(#4370), E-cadherin(#3195), N-cadherin(#13116), Vimentin(#5741), ZEB1(#3396), Snail(#3879), and HRP-conjugated goat anti-rabbit antibodies were purchased from Cell Signaling Technology (Beverly, USA). TFAP2A (Ab108311) were purchased from Abcam (Cambridge, UK). Anti-PCNA(BM0104) and anti-Ki67(PB9026) antibodies were purchased from Boster Bioengineering Co., Ltd. (Wuhan, China). The HRP-conjugated GAPDH (HRP-60004), and β-actin (HRP-66009) antibodies were obtained from Proteintech (Chicago, USA). The HRP-conjugated goat anti-mouse IgG were purchased from ZSGB-BIO (Beijing, China).

### EdU assay

Cells were seeded in 96-well plates. After 24 h, the cells were incubated with EdU solution for 2 h. The medium was discarded. Cells were fixed with 4% paraformaldehyde after washing with PBS. Finally, the cells were sequentially stained with Apollo® 567 fluorescent dye and Hoechst 33342, and photographed under the fluorescence microscope. EdU positive cell ratio was calculated to evaluate cell proliferation ability.

### MTS assay

Cells were seeded in triplicates at a density of 1 × 10^3^ cells per well in a 96-well plate, with each well containing 100 μl of medium. After 24, 48, 72, 96, and 120 h of incubation, cell proliferation was assessed using the Cell Titer 96® Aqueous One Solution Cell Proliferation Assay (Promega, Madison, WI, USA), following the manufacturer’s protocol. Briefly, 20 μl of MTS/PMS solution was added to each well and incubated for an additional 1–4 h at 37 °C in a humidified environment with 5% CO_2_. The absorbance at 490 nm was measured using an ELISA plate reader to quantify cell proliferation. At least triplicate readings were recorded for each time point.

### Colony formation assay

Cells were seeded at a density of 1 × 10^3^ cells per well in a 6-well or 12-well plate for approximately 2 weeks. After washing the plate twice with PBS, ice-cold methanol was added for 15 min to fix the cells. Crystal violet staining was performed for 30 min followed by rinsing with water and air-drying the plate upside down. Cell colonies were observed, photographed, and the number of cells in each clone was calculated.

### Transwell assay

24-well transmembrane chambers, either pre-coated or uncoated with matrix gel, were utilized. The lower chamber was filled with 650 μL of RPMI 1640 culture medium supplemented with 20% fetal bovine serum. In the upper chamber, 2 × 10^4^ cells (for migration assay) or 4 × 10^4^ cells (for invasion assay) of MDA-MB-231 or BT-549 cells in serum-free RPMI 1640 medium were added. Following a 24-h (for migration assay) or 48-h (for invasion assay) incubation period, cells that migrated or invaded into the lower chambers were fixed with methanol, stained with crystal violet and counted.

### Wound healing assay

Cells were plated in 6-well plates to achieve near-complete cell fusion within 24 h. The cell layer was gently scratched using the tip of a 10 μl pipette, and the medium was replaced with PBS. A reference mark was made at the bottom of the 6-well plate to indicate the initial scratch location. Images of the scratch were captured under a microscope. The PBS was then discarded and replaced with fresh medium to resume culture. Microscopic images of the same area were taken at 12-h or 24-h intervals to monitor the progression of the scratch closure.

### Xenograft mouse model

MDA-MB-231 cells, with or without miR-8072 overexpression, were subcutaneously injected into 4-week-old female BALB/c nude mice maintained under SPF conditions. Each injection consisted of 5 × 10^6^ cells suspended in 0.1 ml of PBS per mouse. The length (L) and width (W) of each resulting tumor were measured every 7 days using calipers. Tumor volume was calculated using the formula: L × W^2^ × 0.52. After five weeks of inoculation, the mice were euthanized, and the tumors were excised, weighed, and photographed for further analysis. The maximum diameter in any dimension of the tumor masses measured is less than 1.5 cm. All animal experiments were conducted following the institutional ethical guidelines on animal care and approved by the Ethics Committee of Ningxia Medical University.

### Immunohistochemistry staining and western blot analysis

Immunohistochemistry staining and western blot analysis were conducted following previously established protocols, as described in previous studies [26].

### Quantitative real-time PCR

Total RNA was extracted from the cells using Trizol reagent. 1 μg of each RNA sample was reverse transcribed using the PrimeScript™ RT Master Mix kit (#RR036A, TaKaRa, Japan). Real-time fluorescence quantitative PCR experiments were then performed using SYBR® Premix Ex Taq™ II kit (#RR820A, TaKaRa, Japan). Primer sequences used in the experiment includes *TFAP2A*-forward: 5′-AAGTCAATCTCCCTACACGAG-3′, *TFAP2A*-reverse: 5′-GGAGTAAGGATCTTGCGACTGG-3′; *SNAI1*-forward: 5′-TCGGAAGCCTAACTACAGCGA-3′, *SNAI1*-reverse: 5′-AGATGAGCATTGGCAGCGAG-3′; *GAPDH*-forward: 5′-GGACTCATGACCACAGTCCA-3′, *GAPDH*-reverse: 5′-CCAGTAGAGGCAGGGATGAT-3′. GAPDH was used as an internal control to normalize the gene expression levels. The data were analyzed using the 2^−ΔΔCt^ formula, which allows for relative quantification of target gene expression levels.

### Luciferase reporter assay

To confirm TFAP2A as the target gene of miR-8072, the 3′UTR sequence of TFAP2A was inserted into the pmirGLO vector (Promega, Madison, WI, USA) to generate the wild-type luciferase reporter vector, designated as TFAP2A-WT. Additionally, a mutant vector was constructed with mutations introduced into the 3′UTR region at the binding site of miR-8072, named TFAP2A-mut. miR-8072 mimics (or mimics control) and either TFAP2A-WT or TFAP2A-mut were co-transfected into HEK293T cells using Lipofectamine 2000. After 48 h, luciferase activity in the transfected cells was measured using a dual luciferase reporter gene assay kit (Promega, Madison, WI, USA) according to the manufacturer’s instruction.

To validate the transcriptional regulation of *SNAI1* by TFAP2A, a luciferase reporter plasmid containing the promoter sequences of SNAI1 (− 1977 to + 23) was constructed. HEK293T cells were co-transfected with a TFAP2A-overexpressing vector (or an empty vector as control) and the luciferase reporter plasmid using Lipofectamine 2000 transfection reagent. After 48 h, the firefly luciferase activity was assessed in the transfected cells using the Firefly Luciferase Reporter Gene Assay Kit (Beyotime Biotechnology, Shanghai, China) following the manufacturer’s instruction.

### Survival analysis

The association between miR-8072 expression levels and overall survival of breast cancer patients was investigated using the Kaplan–Meier plotter database (https://kmplot.com/analysis/index.php?p=service&cancer=breast_mirna) [27], an online survival analysis platform. The association between TFAP2A expression levels and overall survival of breast cancer patients was analyzed using Kaplan–Meier plotter database (http://kmplot.com/analysis/index.php?p=service&cancer=breast_rnaseq_gse96058) [28].

### Statistical analysis

Statistical analysis was conducted using GraphPad Prism version 8.0 software (GraphPad, San Diego, CA, USA). The data are presented as mean ± standard deviation (SD) from at least three independent biological experiments or samples. The two-tailed Student’s t-test for independent samples was employed to determine the significance of differences between two groups. A *P*-value less than 0.05 was considered statistically significant.

## Results

### miR-8072 hinders TNBC tumor progression in vivo and correlates with better prognosis in TNBC

**Fig. 1:**
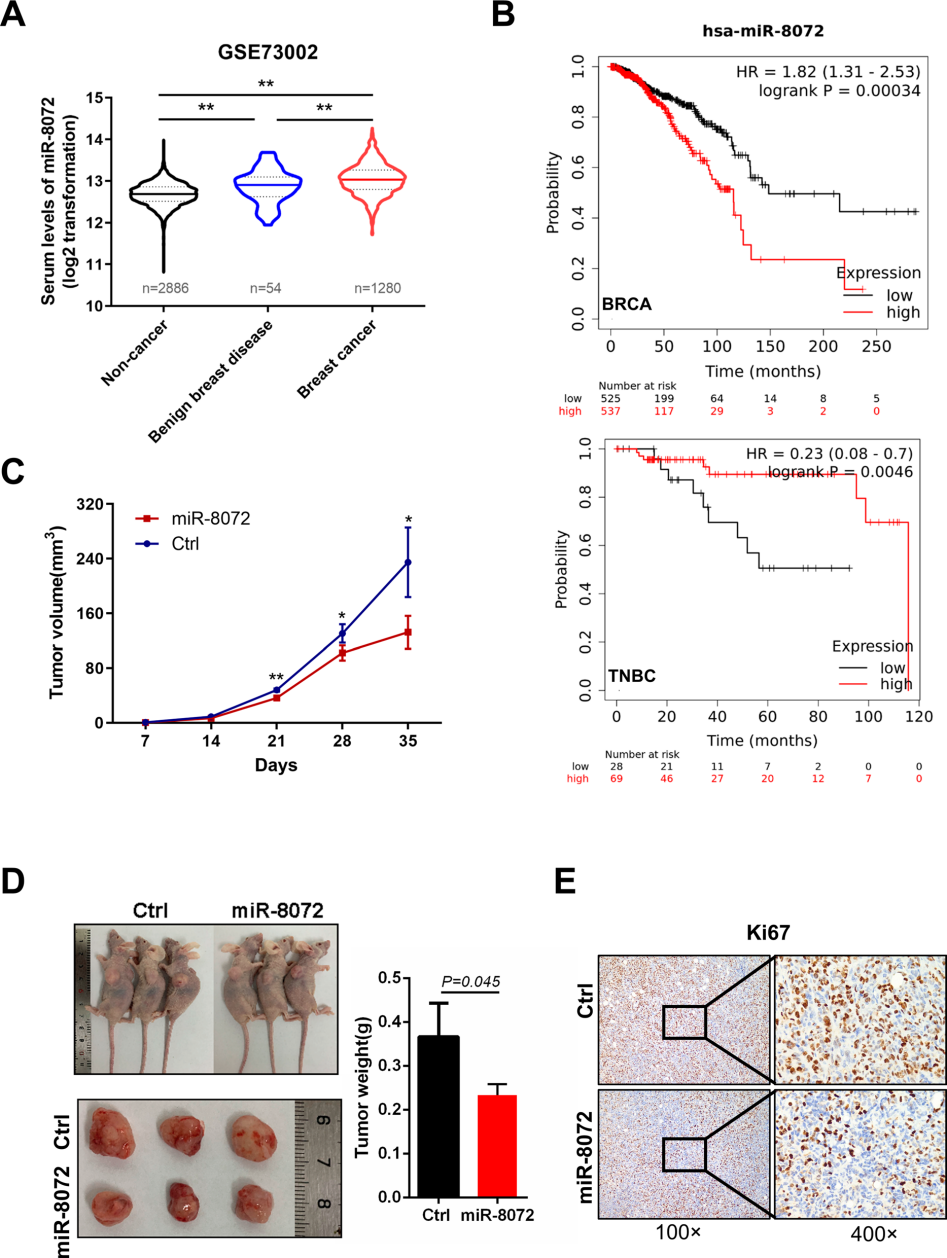
miR-8072 suppresses TNBC tumor progression and predicts favorable prognosis. (**A**) Serum levels of miR-8072 in breast cancer, benign breast disease, and non-cancer samples were analyzed using data from GEO (GSE73002) (**P < 0.01)**. B** The relationships between miR-8072 level and overall survival (OS) in breast cancer patients were analyzed using public database Kaplan–Meier plotter (https://kmplot.com/analysis/index.php?p=service&cancer=breast_mirna). 1062 breast cancer patients were included, 525 cases were in miR-8072 low-expression cohort, and 537 cases were in miR-8072 high-expression cohort. The median survival for the high-expression cohort of miR-8072 is 115.4 months, while it is 148.53 months for the low-expression cohort of miR-8072 (upper panel). 97 TNBC patients were included for analysis. Among them, 28 cases were in miR-8072 low-expression cohort, and 69 cases were in miR-8072 high-expression cohort. The upper quartile survival for cohorts with high-expression of miR-8072 is 98.83 months, while it is 36.43 months for cohorts with low-expression of miR-8072 (lower panel). **C** MDA-MB-231 cells with stably overexpressed miR-8072 or control (Ctrl) cells were subcutaneously implanted in nude mice, tumor volume were measured every 7 days. **D** Tumor nodules were collected and weighed 35 days later after implantation. **E** Expression of Ki67 in the tumor nodules was detected by immunohistochemistry

Previously, in a comparative analysis of constructed MDA-MB-231 cell lines with varying proliferation and migration capacities, we observed a significant and pronounced upregulation of miR-8072 in cells exhibiting reduced proliferation and migration abilities (data not shown), which promoting us to explore whether miR-8072 is a crucial tumor-suppressor in breast cancer. To address it, we initially analyzed the levels of miR-8072 between breast cancer patients and non-cancer controls using a GEO dataset (GSE73002). Unexpectedly, compared with non-cancer controls, serum levels of miR-8072 was increased in breast cancer patients (Fig. 1A). Furthermore, we also found a negative correlation between miR-8072 expression and the overall survival (OS) of breast cancer patients in the Kaplan–Meier plotter database (Fig. 1B, upper panel), which is not consistent with our original speculation. Given that the MDA-MB-231 cell line represents a triple negative breast cancer (TNBC) cell line, we were intrigued to investigate whether miR-8072 exhibits unique functionality in TNBC. As shown in Fig. 1B (lower panel), TNBC patients exhibiting elevated miR-8072 expression levels demonstrated a significantly prolonged overall survival (OS) compared to those with low miR-8072 expression. For further validation, we investigated the effect of miR-8072 on the in vivo growth of MDA-MB-231 cells. Remarkably, the tumor masses derived from MDA-MB-231 cells with overexpressed miR-8072 exhibited significantly diminished size, volume, and weight when compared to the control cells (Fig. 1C and D). Furthermore, there was a notable decrease in the expression of Ki-67, a specific marker for cell proliferation, in the tumor masses formed from cells overexpressing miR-8072 (Fig. 1E). Collectively, these findings substantiate the tumor-suppressive function of miR-8072 in TNBC, highlighting its utility as a valuable prognostic indicator indicative of a favorable outcome in TNBC.

### miR-8072 inhibits activation of AKT/ERK signaling and suppresses proliferation of TNBC cells

**Fig. 2:**
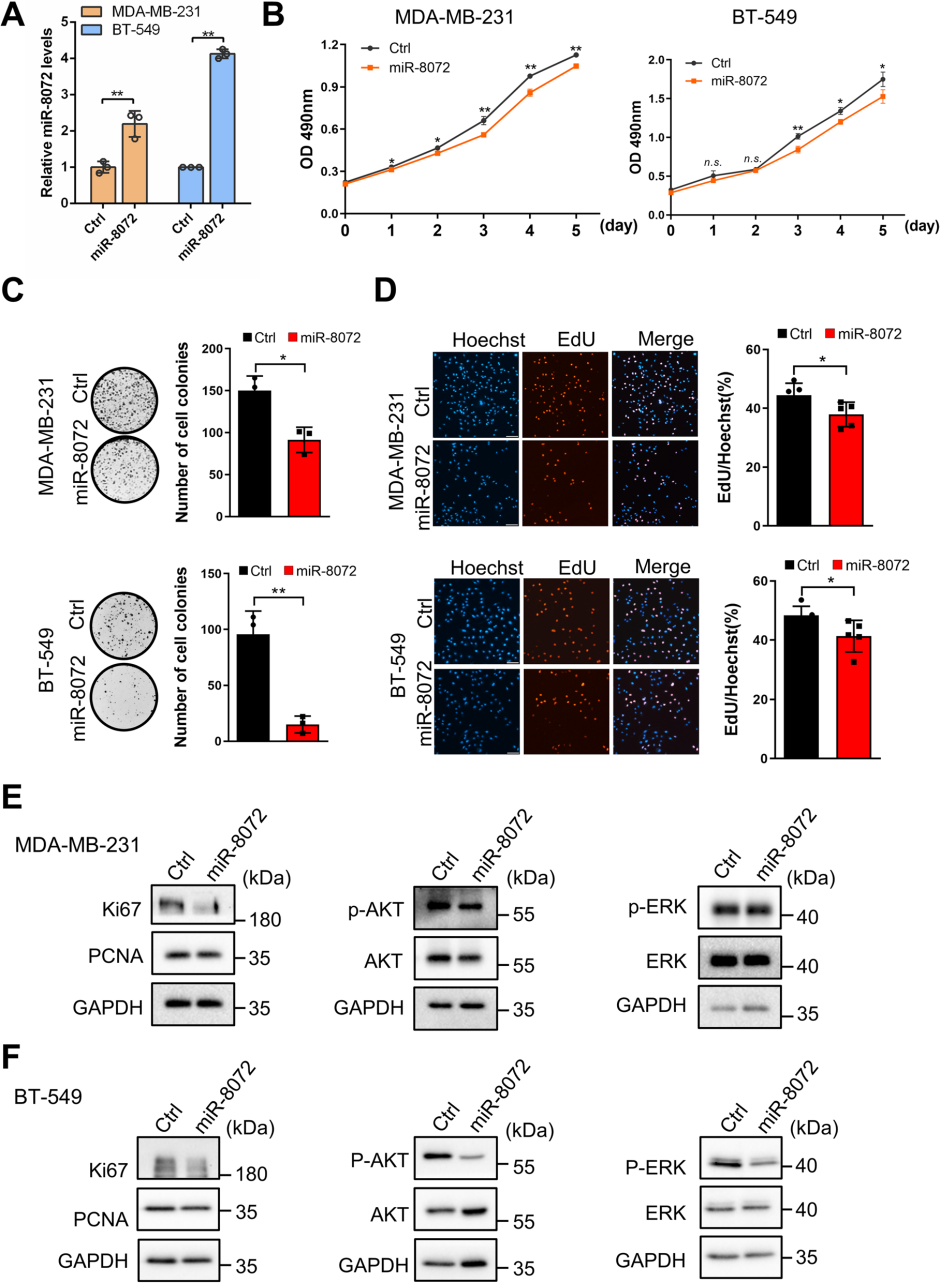
miR-8072 inhibits activation of AKT/ERK signaling and suppresses proliferation of TNBC cells. **A** Detection of miR-8072 overexpression in TNBC cells by qRT-PCR. Cell proliferation was measured by **B** MTS assay, **C** Colony formation assay, and **D** EdU assay in MDA-MB-231 cells and BT-549 cells after miR-8072 was overexpressed. **E** Western blot analysis depicting the expression levels of proliferation-related protein levels or activation status in MDA-MB-231 cells and **F** BT-549 cells after miR-8072 was overexpressed

To investigate the impact of miR-8072 on the proliferative capacity of TNBC cells, we overexpressed or functionally inhibited miR-8072 in MDA-MB-231 and BT-549 cell lines. Subsequently, we assessed the proliferative potential of TNBC cells using various methodologies. The results obtained from the MTS assay demonstrated that miR-8072 overexpression significantly inhibited the growth of both MDA-MB-231 and BT-549 cells compared to the control cells (Fig. 2A, B). Whereas, functional inhibition of miR-8072 promoted the growth of MDA-MB-231 cells (Supplementary Fig. 1A). The colony formation assay revealed a substantial reduction in the number of colonies formed by TNBC cells overexpressing miR-8072 (Fig. 2C). Conversely, functional inhibition of miR-8072 led to an increase in colony formation in MDA-MB-231 cells (Supplementary Fig. 1B).

To further validate the impact of miR-8072 on the proliferation ability of TNBC cells, we conducted EdU experiments. The results clearly indicated a significant decrease in the number of EdU-positive cells in both MDA-MB-231 and BT-549 cells overexpressing miR-8072 compared to the control cells (Fig. 2D). Conversely, functional inhibition of miR-8072 in MDA-MB-231 cells exhibited an opposite phenomenon (Supplementary Fig. 1C). Furthermore, at the molecular level, we found the downregulation of Ki67 and PCNA proteins and decreased activation of AKT and ERK signaling, which are associated with proliferation, following miR-8072 overexpression in both MDA-MB-231 and BT-549 cells (Fig. 2E and F). Conversely, functional inhibition of miR-8072 in MDA-MB-231 cells upregulated the expression levels of proliferation-related markers and activated AKT and ERK signaling (Supplementary Fig. 1D). 

Taken together, these findings strongly indicate that miR-8072 exhibits inhibitory effects on the proliferation ability of TNBC cells.

### miR-8072 suppresses EMT progression and migration of TNBC cells

**Fig. 3:**
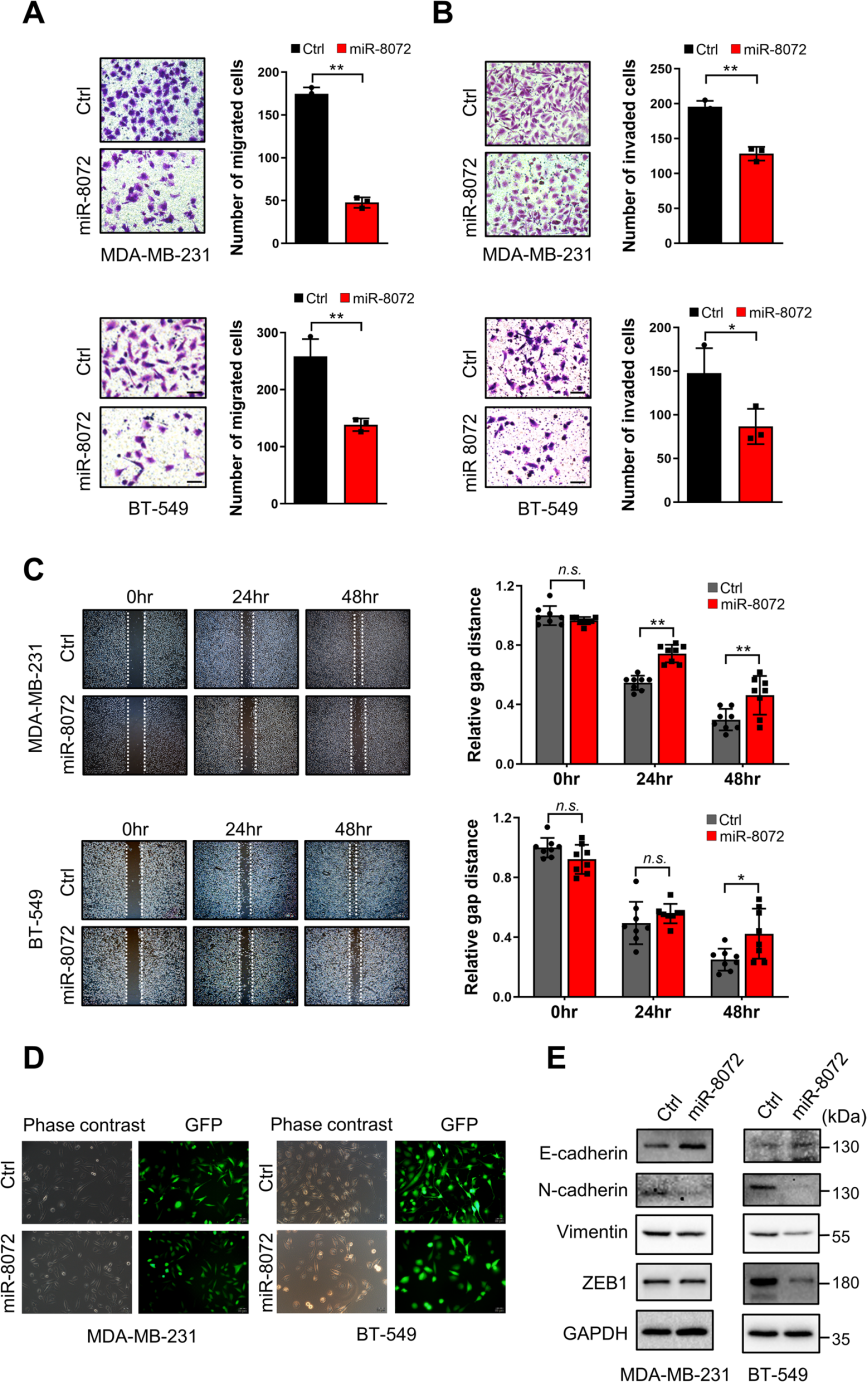
miR-8072 suppresses migration and invasion of TNBC cells via inhibiting EMT Progression. **A** Transwell assay demonstrating the effect of miR-8072 overexpression on the migration and **B** invasion abilities of MDA-MB-231 and BT-549 cells after miR-8072 was overexpressed. Representative images of migrated and invaded cells through the porous membrane are shown. **C** Scratch wound healing assay showing the migratory capacity of MDA-MB-231 and BT-549 cells with miR-8072 overexpression or control. Representative images were captured at 0, 24 and 48 h post-scratch. **D** Representative images depicting cellular morphology of MDA-MB-231 and BT-549 cells with or without miR-8072 overexpression, as observed under a fluorescence microscope. **E** Western blot analysis depicting the expression levels of E-cadherin, N-cadherin, Vimentin, and ZEB1 proteins in MDA-MB-231 and BT-549 cells with and without miR-8072 overexpression

As a highly invasive and metastatic subtype of breast cancer, TNBC is often associated with poor prognosis and lacks effective therapeutic strategies targeting its invasion and metastasis. Since miR-8072 is positively correlated with favorable prognosis of TNBC, we then investigated whether miR-8072 could affect migration and invasion abilities of TNBC cells. The transwell assay demonstrated that overexpression of miR-8072 led to a significant decrease in both migration and invasion abilities of MDA-MB-231 and BT-549 cells at the vertical level, compared to the control group (Fig. 3A and B). Conversely, functional inhibition of miR-8072 in MDA-MB-231 cells significantly increased their migration and invasion abilities at the vertical level (Supplementary Fig. 2A and B). Moreover, the results obtained from the scratch wound healing assay revealed a significant reduction in the horizontal migration ability of MDA-MB-231 and BT-549 cells overexpressing miR-8072 compared to control cells (Fig. 3C). Conversely, functional inhibition of miR-8072 enhanced the horizontal migration ability of MDA-MB-231 cells (Supplementary Fig. 2C).

EMT is a critical process in tumor progression and metastasis. TNBC is particularly prone to undergoing EMT-associated molecular changes, further emphasizing the significance of this process in driving its aggressive behavior and metastatic potential. Therefore, we proceeded to investigate changes in EMT process following modulation of miR-8072 expression or function. As shown in Fig. 3D, cells overexpressing miR-8072 exhibited a more compact and organized epithelial-like morphology compared to control cells in both MDA-MB-231 and BT-549 cell lines. At the molecular level, overexpression of miR-8072 demonstrated a significant upregulation in E-cadherin expression, which is a hallmark of epithelial phenotype, while concurrently causing a downregulation in the expression levels of markers associated with the mesenchymal phenotype, including N-cadherin, Vimentin, and ZEB1, in both MDA-MB-231 and BT-549 cells (Fig. 3E). Conversely, functional inhibition of miR-8072 in MDA-MB-231 cells exhibited contrasting effects, signifying a shift towards a more mesenchymal-like phenotype after inhibiting miR-8072 in TNBC cells. (Supplementary Fig. 2D). Collectively, these findings highlight the crucial involvement of miR-8072 in suppressing EMT-related events and further contributes to the suppression of cellular plasticity and invasive capabilities, ultimately inhibiting the migratory and invasive behavior of TNBC cells.

### *TFAP2A* is a target gene of miR-8072 associated with unfavorable prognosis in TNBC

**Fig. 4:**
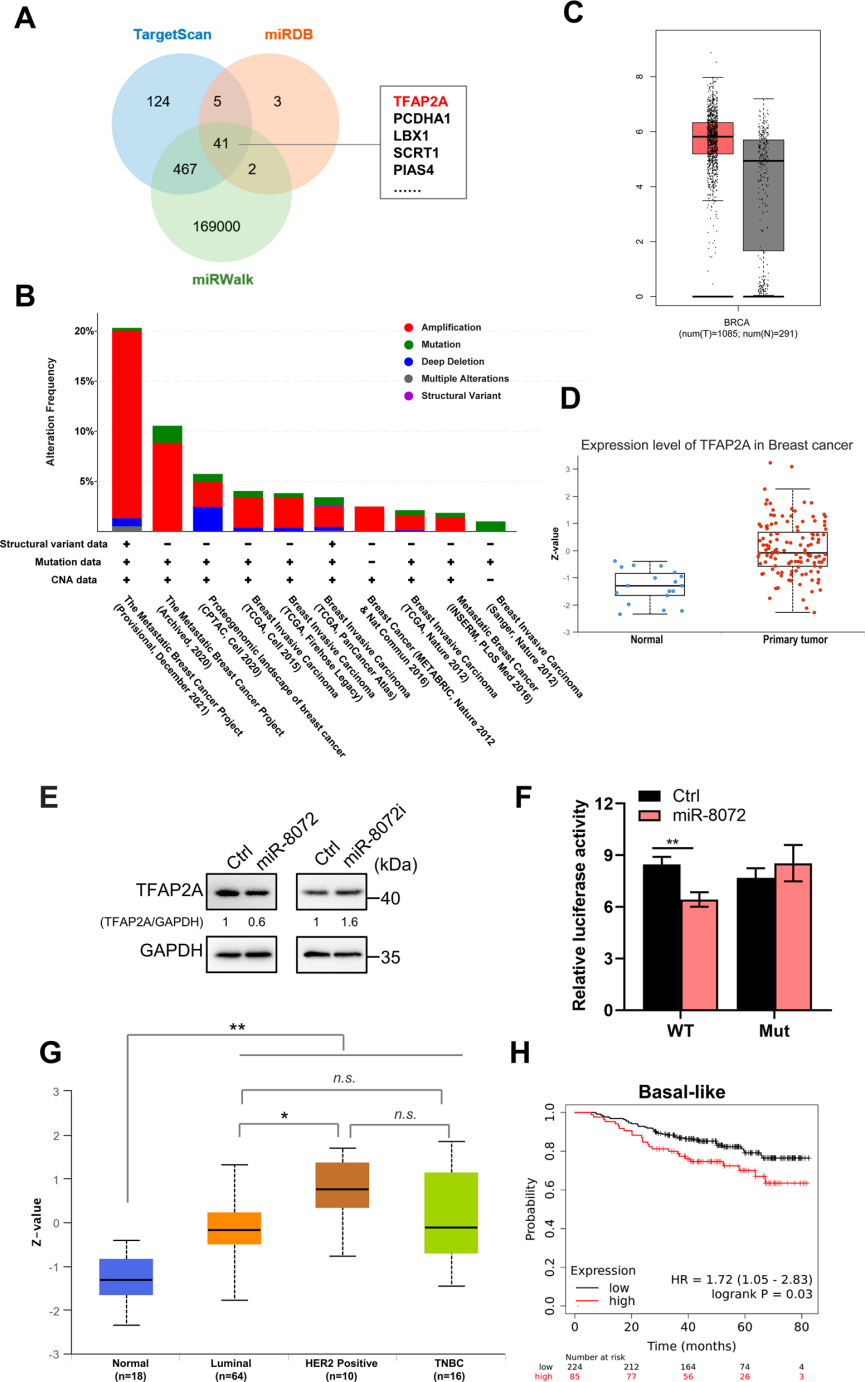
*TFAP2A* is a target gene of miR-8072 and is associated with unfavorable prognosis in TNBC. **A** Venn diagram depicting common putative targets of miR-8072 through bioinformatics analysis using the miRWalk, TargetScan, and miRDB databases. **B** *TFAP2A* gene alteration across different breast cancer cohorts in the TCGA database. **C** Comparison of *TFAP2A* mRNA expression between breast cancer and normal tissues using the GEPIA database. **D** Comparison of TFAP2A protein levels between breast cancer and normal tissues using the UALCAN database. **E** Evaluation of TFAP2A protein levels upon modulation of miR-8072 expression in BT-549 cells. **F** Luciferase reporter assay confirming a direct interaction between miR-8072 and the 3′UTR of *TFAP2A*. **G** Analysis of TFAP2A protein expression in different breast cancer subtypes. **H** Survival analysis indicating a negative correlation between TFAP2A expression and overall survival in basal-like breast cancer

To gain insights into the mechanisms underlying the anti-tumor effects of miR-8072 in TNBC, we probed to identify the target gene regulated by miR-8072 during this process. Through a comprehensive bioinformatics analysis utilizing multiple databases, *TFAP2A* emerged as a particularly intriguing candidate due to its crucial role in maintaining the basal-like phenotype of breast cancer [9] (Fig. 4A). In ER-positive breast cancer, a few studies have shown that TFAP2A promotes tumor progression [11, 12]. However, the involvement of TFAP2A in TNBC remains largely unexplored. Upon analyzing the genomic alterations of *TFAP2A* in breast cancer using data from TCGA available at http://cbioportal.org, we observed that the most common alteration for *TFAP2A* is gene amplification (Fig. 4B). This finding further supports the notion that *TFAP2A* acts as an oncogene in breast cancer. Consistently, comparing with corresponding normal tissues, *TFAP2A* mRNA and protein levels are upregulated in breast cancer (Fig. 4C and D). To validate the regulatory relationship between miR-8072 and *TFAP2A*, we firstly evaluated the impact of miR-8072 on *TFAP2A* expression levels, which showed that TFAP2A protein level is reduced after overexpression of miR-8072, but elevated when miR-8072 was functionally inhibited (Fig. 4E, Supplementary Fig. 3A). Luciferase reporter assay further confirmed a direct interaction between miR-8072 and the 3′UTR of *TFAP2A*. Specifically, transfection of miR-8072 mimics significantly suppressed the relative luciferase activity of the TFAP2A-WT reporter compared to control mimics. Importantly, this inhibitory effect was abolished when the nucleotides of *TFAP2A* 3′UTR binding to the seed sequence of miR-8072 was mutated (Fig. 4F, Supplementary Fig. 3B).

To specifically explore the potential role of TFAP2A in TNBC, we investigated the TFAP2A protein levels in different subtypes of breast cancer. As shown in Fig. 4G, HER2 positive breast cancer has a highest expression of TFAP2A, which is consistent with the reports that TFAP2A could regulate HER2 expression [29]. TNBC also has a relative high expression of TFAP2A. Although, TFAP2A is highly expressed in luminal breast cancer, SUMOylation endowed to TFAP2A hinders its transcriptional activation on luminal-type associated genes [9]. Furthermore, we analyzed association between TFAP2A expression and prognosis of breast cancer using survival analysis. Interestingly, among all the breast cancer subtypes, we observed a negative correlation between TFAP2A expression and patients’ overall survival time in basal-like breast cancer, whereas a positive correlation was observed in luminal B breast cancer (Fig. 4H, Supplementary Fig. 3C).

Taken together, the aforementioned results demonstrate *TFAP2A* is a significant target gene of miR-8072 and potentially functions as an oncogene in TNBC.

### The tumor-promoting role of TFAP2A and its functional interplay with miR-8072

**Fig. 5:**
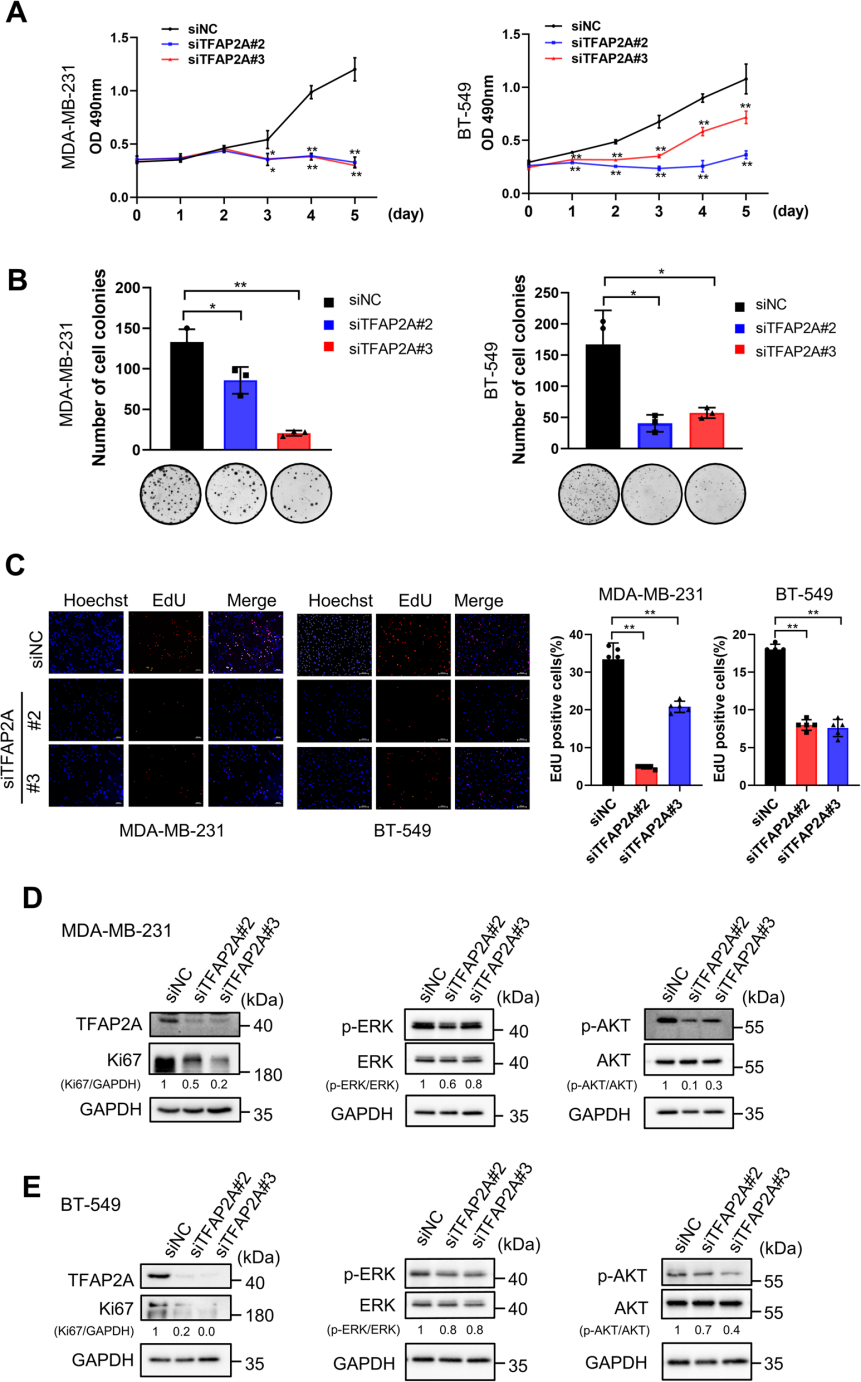
Silencing TFAP2A inhibits proliferation of TNBC cells. Expression of TFAP2A was knocked down by siRNAs in MDA-MB-231 and BT-549 cells, cell proliferation was assessed by **A** MTS assay, **B** Colony formation assay, and **C** EdU assay. **D** Western blot analysis showing the expression levels of Ki67, TFAP2A, and the phosphorylation levels of ERK and AKT in MDA-MB-231 cells and **E** BT-549 cells with or without TFAP2A knockdown

Building upon our previous findings regarding the anti-tumor effects of miR-8072, we conducted further investigations to validate the involvement of TFAP2A in TNBC. Silencing TFAP2A using siRNAs in MDA-MB-231 and BT-549 cells significantly reduced cell proliferation, as observed through MTS assay, colony formation assay, and EdU assay (Fig. 5A–C, Supplementary Fig. 4). Western blot analysis further supported these results, revealing downregulation of proliferation-related proteins Ki67, as well as inhibited activation of AKT and ERK signaling, upon TFAP2A silencing (Fig. 5D, E).

**Fig. 6:**
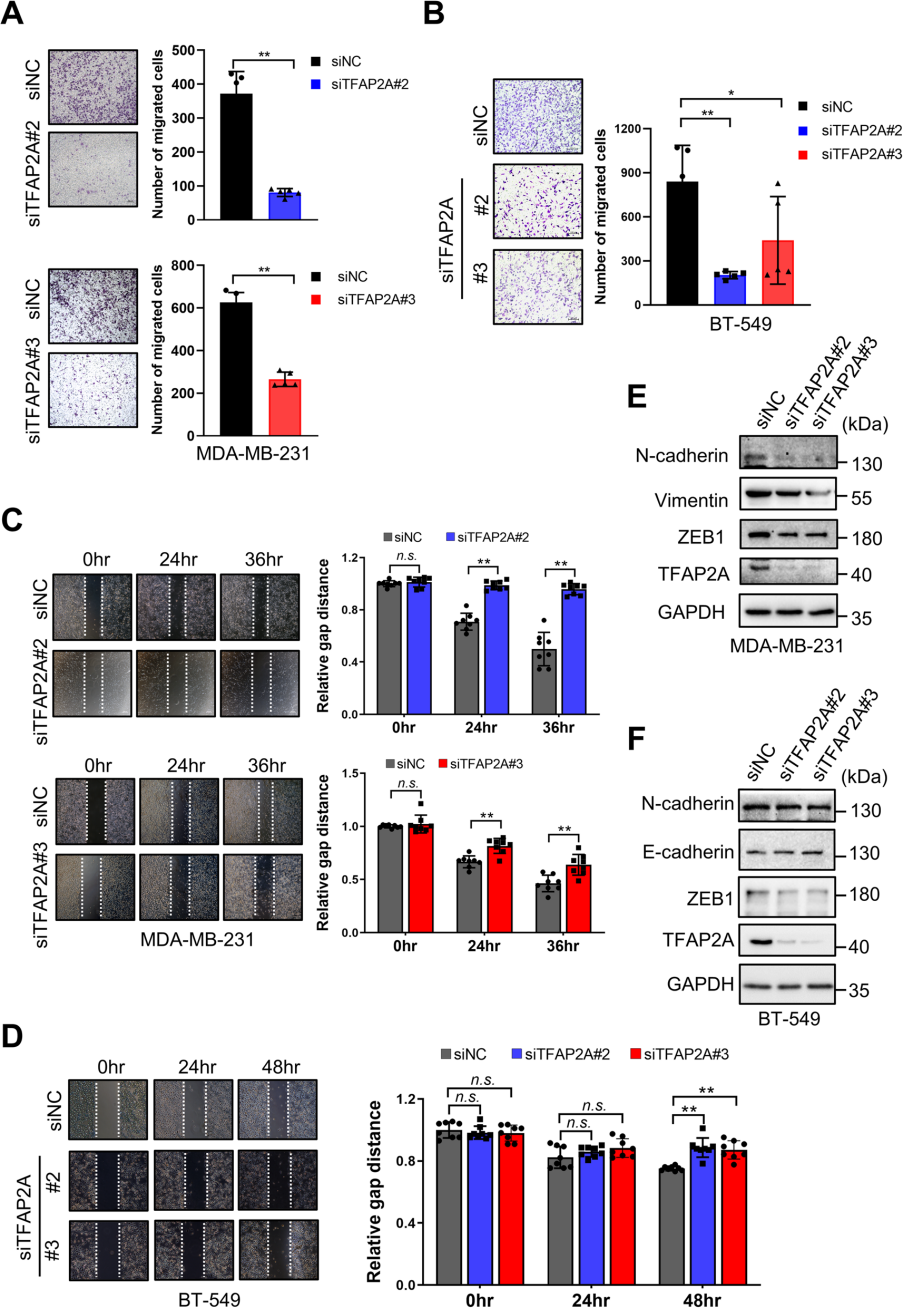
Silencing TFAP2A suppresses migration of TNBC cells. Cell migration of **A** MDA-MB-231 and **B** BT-549 cells with or without TFAP2A knockdown were investigated by transwell assay. Scratch wound healing assay was performed to investigate cell migration of **C** MDA-MB-231 and **D** BT-549 cells with or without TFAP2A knockdown. Western blot analysis depicting the expression levels of EMT-related proteins in **E** MDA-MB-231 and **F** BT-549 cells with or without TFAP2A knockdown

In terms of cell migration, both wound healing and transwell assays demonstrated a significant decrease in the migratory ability of TFAP2A-silenced MDA-MB-231 and BT-549 cells, compared to control cells (Fig. 6A–D). Western blot analysis revealed changes in protein expression levels associated with EMT, with upregulation of E-cadherin and downregulation of N-cadherin, Vimentin, and ZEB1 upon TFAP2A knockdown in MDA-MB-231 and BT-549 cells, indicating a suppression of the EMT process (Fig. 6E and F).

**Fig. 7:**
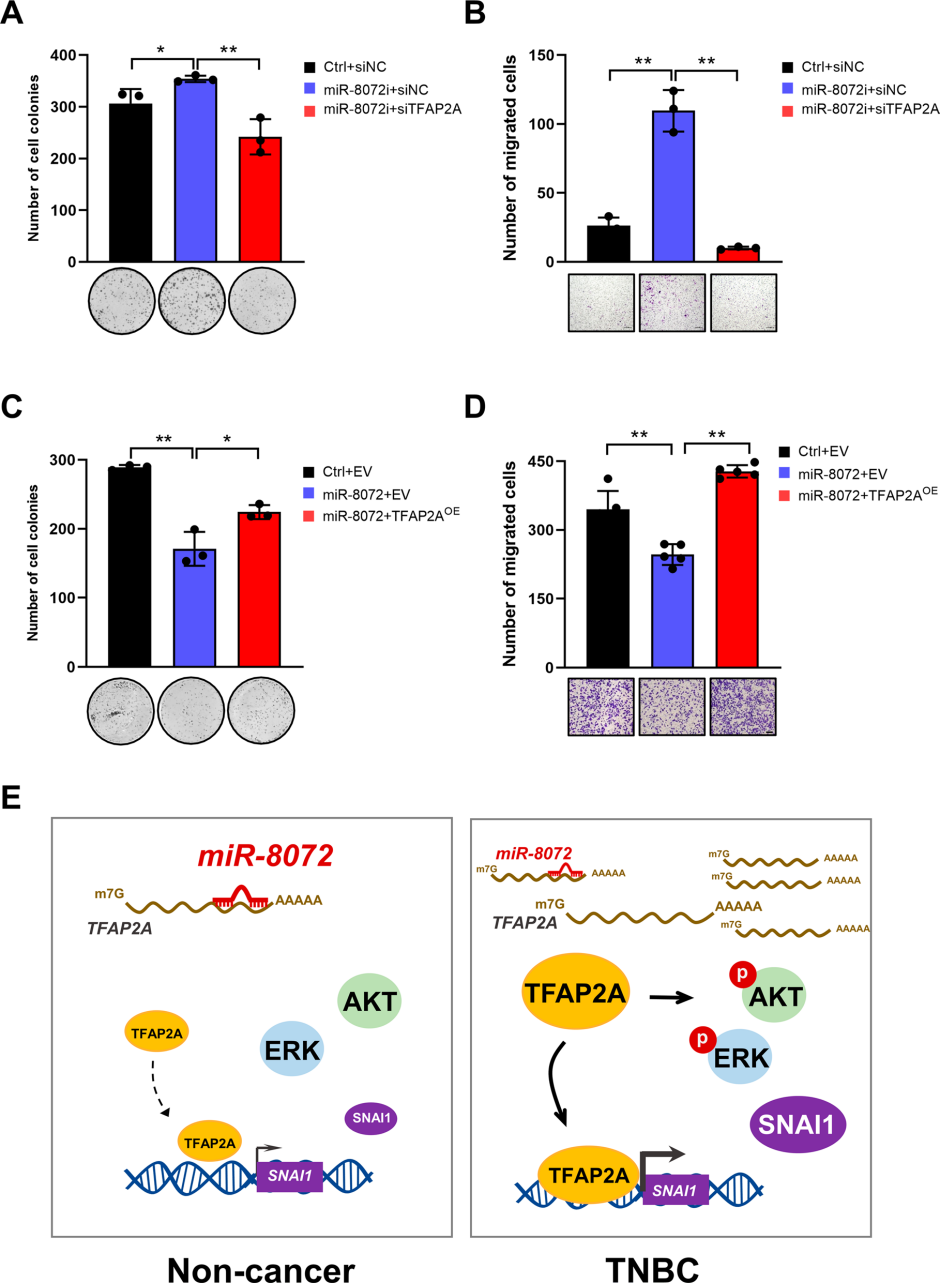
TFAP2A mediates tumor-suppressive effects of miR-8072 in TNBC cells. MDA-MB-231 cells were transfected with siTFAP2A or siNC in the presence or absence of miR-8072 functional inhibition, subsequently, **A** colony formation assay was performed to assess cell proliferation ability, and **B** transwell assay was used to evaluate cell migration. **C** Colony formation assay and **D** transwell assay were conducted to evaluate the influence of TFAP2A overexpression on the proliferation and migration abilities of miR-8072-overexpressing BT-549 cells. **E** Working model illustrating the tumor-suppressive effect of miR-8072 through TFAP2A downregulation-mediated transcriptional inhibition of *SNAI1* in TNBC cells

In order to determine the involvement of TFAP2A in miR-8072-mediated action on proliferation and migration of TNBC cells, we performed a rescue assay. MDA-MB-231 cells were transfected with siTFAP2A or siNC in the presence or absence of miR-8072 functional inhibition to investigate their interplay on cell proliferation and migration. The results indicated functional inhibition of miR-8072 enhanced the proliferation and migration capabilities of MDA-MB-231 cells; silencing TFAP2A could mitigate the stimulatory effects of miR-8072 inhibition on the proliferation and migration of the cells (Fig. 7A and B, supplementary Fig. 5A). Similarly, the inhibitory impact of miR-8072 on the proliferation and migration of BT-549 cells could be alleviated by TFAP2A overexpression (Fig. 7C and D, supplementary Fig. 5B).

Collectively, the aforementioned results not only provide compelling evidence for a tumor-promoting role of TFAP2A in TNBC, but also indicate that TFAP2A is intricately associated with miR-8072-mediated suppression on proliferation and migration of TNBC cells, highlighting the continuous functional cascade initiated by miR-8072 in suppressing tumor progression.

### Transcriptional activation of *SNAI1* by TFAP2A is suppressed by miR-8072

**Fig. 8:**
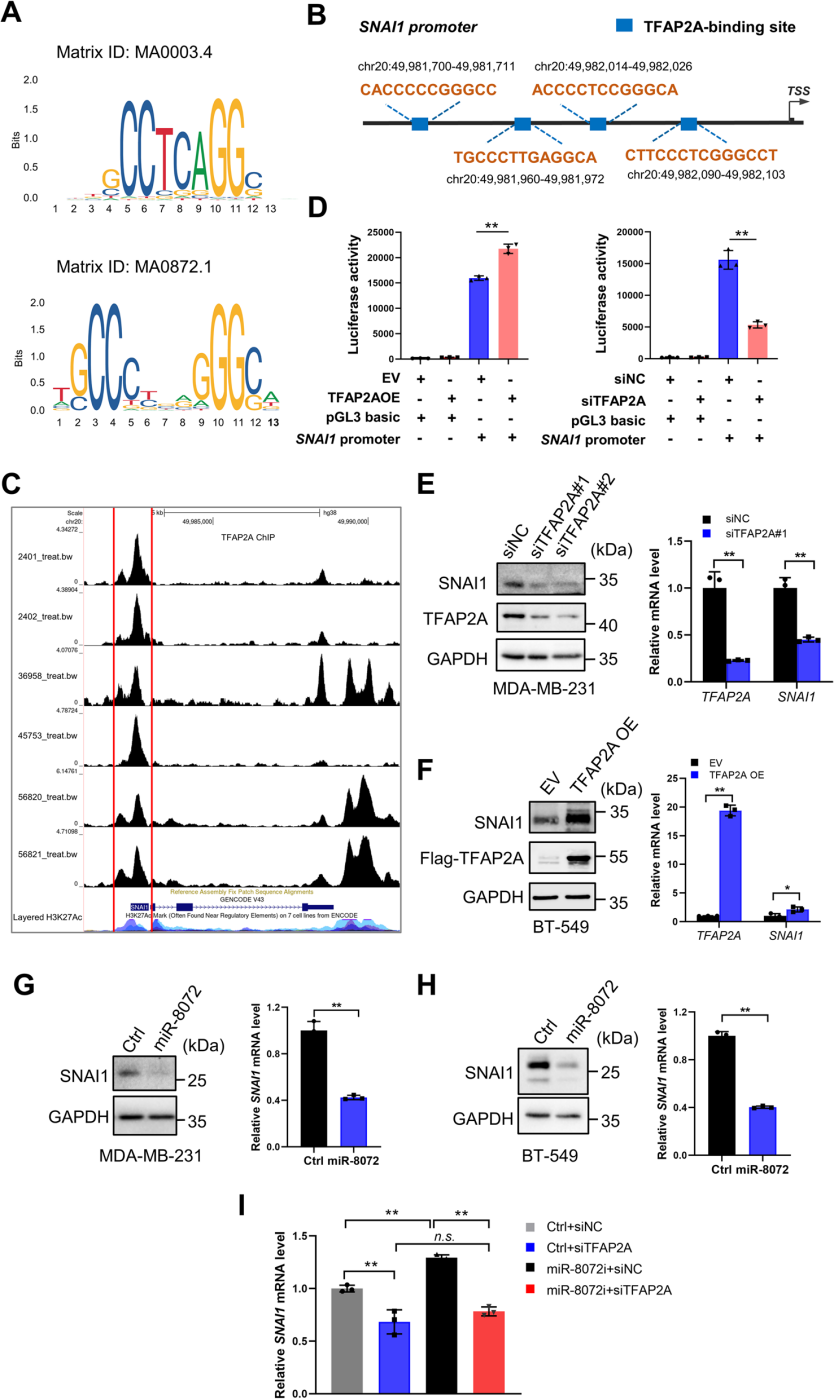
Transcription of *SNAI1* is repressed by miR-8072/TFAP2A axis. **A** DNA sequence motif recognized by TFAP2A were obtained in JASPAR database. **B** Schematic showing the putative TFAP2A-binding sites within *SNAI1* promoter region. **C** TFAP2A enrichment on the *SNAI1* promoter were analyzed using public ChIP-Seq database (http://dc2.cistrome.org). **D** Luciferase reporter assay demonstrating the transcriptional regulation of *SNAI1* by TFAP2A. **E** mRNA and protein expression levels of *SNAI1* were measure by qRT-PCR and western blot, after TFAP2A was silenced in MDA-MB-231 cells or **F** overexpressed in BT-549 cells. **G** mRNA and protein expression levels of *SNAI1* were measure by qRT-PCR and western blot after miR-8072 was overexpressed in MDA-MB-231 cells or **H** BT-549 cells. **I** *SNAI1* mRNA levels were determined in MDA-MB-231 cells after transfection with siTFAP2A or siNC in the presence or absence of miR-8072 functional inhibition

Next, we asked how TFAP2A regulates TNBC proliferation and migration. Given TFAP2A is a transcription factor, our focus was on identifying potential target genes that are transcriptionally regulated by TFAP2A. Using the cistrome data browser, we found *SNAI1*, a critical player in EMT and cell proliferation regulation, is one of the top ten putative targets of TFAP2A based on a ChIP-seq data (Supplementary Fig. 6A). The DNA sequence motif recognized by TFAP2A is depicted in Fig. 8A. Furthermore, we found the presence of four potential TFAP2A binding sites within the *SNAI1* promoter region (Fig. 8B). In addition, by analyzing several ChIP-Seq data sets, we observed a noticeable enrichment of TFAP2A on the promoter of *SNAI1*, which is colocalized with H3K27Ac peaks, a marker of transcriptional activation, within *SNAI1* promoter region (Fig. 8C). For further validation, we conducted luciferase reporter assay to validate the transcriptional regulation of *SNAI1* by TFAP2A. The results showed that co-transfection of a TFAP2A-overexpressing vector with a firefly luciferase reporter vector containing *SNAI1* promoter could obviously enhance luciferase activity in HEK293T cells when compared to cells transfected with empty vector. Conversely, silencing TFAP2A exhibited an opposite effect (Fig. 8D), providing compelling evidence that *SNAI1* is a target gene of TFAP2A. Furthermore, the mRNA and protein levels of *SNAI1* were remarkably decreased after silencing of TFAP2A in TNBC cells (Fig. 8E, Supplementary Fig. 6B). Conversely, TFAP2A overexpression led to an upregulation of *SNAI1* mRNA and protein levels (Fig. 8F). Therefore, these results indicate that TFAP2A transcriptionally regulates expression of *SNAI1*.

To further investigate the relationship between *SNAI1* and miR-8072-mediated downregulation of TFAP2A, we assessed the impact of miR-8072 on the expression of *SNAI1* at both the mRNA and protein levels. As shown in Fig. 8G and H, overexpression of miR-8072 led to a suppression of *SNAI1* expression. Crucially, the rescue experiments further revealed that knockdown of TFAP2A attenuated elevation of *SNAI1* expression induced by miR-8072 functional inhibition (Fig. 8I). These results provide additional support for the role of the miR-8072/TFAP2A axis in modulating *SNAI1*.

In conclusion, the above experimental results collectively suggest that TFAP2A downregulation mediates tumor-suppressive effect of miR-8072 in TNBC cells via inhibiting *SNAI1* transcription and EMT progression, as well as activation of AKT and ERK signaling (Fig. 7C). These findings provide further insights into the intricate regulatory mechanisms governing TNBC progression and highlight potential avenues for therapeutic intervention.

## Discussion

As a highly invasive and metastatic subtype of breast cancer, triple-negative breast cancer (TNBC) is often associated with poor prognosis and lacks effective therapeutic strategies targeting its invasion and metastasis. Therefore, exploring the molecular mechanisms regulating TNBC invasiveness and metastasis is of significant importance, and miR-8072 has emerged as a potential regulatory factor of interest in our preliminary screening. Studies on miR-8072 is very limited, and its involvement in disease is not clear. One research reporting that miR-8072 is associated with shorter survival in gastric cancer [30]. We found serum levels of miR-8072 are increased in breast cancer in comparison to non-cancer individuals and benign breast disease, and high expression of miR-8072 is associated with shorten overall survival in breast cancer. Interestingly, in TNBC, miR-8072 appears to serve as a better prognostic indicator; patients with high miR-8072 expression had longer overall survival time. Through further validation, we confirmed miR-8072 functions as a tumor suppressor in TNBC, as indicated by its inhibitory effects on proliferation, migration and invasion of TNBC cells. In light of the recent breakthroughs in nanoparticle-based delivery systems, particularly in the context of miRNAs/siRNAs or compounds, including exosomes, as therapeutic agents for disease treatment [31–33], the potential of miR-8072 as a remarkable and efficacious therapeutic molecule for TNBC holds immense promise.

In our endeavor to unravel the mechanism underlying the inhibitory effect of miR-8072 on tumorigenesis in TNBC, we conducted an analysis to identify potential targets of miR-8072, ultimately identifying TFAP2A as a noteworthy candidate due to its known roles in cancer development and progression, particularly in different subtypes of breast cancer. TFAP2A stands as a prominent member among the five mammalian counterparts belonging to the AP-2 family of transcription factors [34]. Genes harboring AP-2-binding sites within their promoter regions play pivotal roles in critical biological processes, including cell growth and differentiation. Consequently, AP-2 proteins serve as crucial gatekeepers that delicately balance proliferation and differentiation during embryogenesis [34]. Notably, enhanced levels of AP-2 have been observed across diverse types of cancer, further highlighting their significance in tumorigenesis [6, 35, 36]. TFAP2A has been suggested to function as a tumor suppressor in many cancers, but recently, many studies reported the tumor-promoting effects of TFAP2A in different cancer types. For instance, TFAP2A potentiates lung adenocarcinoma metastasis and stimulates angiogenesis in lung cancer cells with acquired resistance to anlotinib [37, 38]; TFAP2A-mediated activation of E2F and EZH2 drives melanoma metastasis [39]. TFAP2A, along with TFAP2B and TFAP2C, are known to be expressed in breast tissue and coordinated the growth and development of the breast via regulation of several breast-related genes such as human epidermal growth factor receptor-2(HER2) and estrogen receptor (ER) [40–43]. In contrast to TFAP2C, extensive research indicates that TFAP2A plays a significant tumor suppressive role in breast tissues, exhibiting functions crucial for growth suppression and maintenance of the differentiated state [43–45]. Recent evidence suggests that TFAP2A may promote the progression of breast cancer [11]. Of note, in luminal breast cancer subtypes, TFAP2A has been found to exhibit reduced transcriptional activity on luminal genes due to its high sumoylation level. However, it plays a critical role in maintaining the basal breast cancer subtype. In the present study, we specifically investigate the role of TFAP2A in TNBC, and found TFAP2A expression is negatively associated with overall survival of TNBC patients; whereas in Luminal B subtype breast cancer, high expression of TFAP2A indicates a favorable outcome. These findings imply a notable divergence in the transcriptional regulatory role and function of TFAP2A across different breast cancer subtypes. Through functional experiments, we found TFAP2A promotes proliferation and migration of TNBC cells, and the tumor-suppressive effects of miR-8072 in TNBC are mediated through post-transcriptional downregulation of TFAP2A.

SNAI1, also known as snail family transcriptional repressor 1 (SNAIL1), plays a crucial role in breast cancer progression and metastasis [18–20, 46]. As a transcriptional repressor, SNAI1 is known for its ability to repress E-cadherin expression and promote the acquisition of a mesenchymal phenotype during EMT, a process that confers increased invasive and migratory properties to cancer cells. In addition, SNAI1 may indirectly influence cell proliferation by modulating various downstream signaling pathways [47, 48]. In this study, we have identified *SNAI1* as a direct target gene of TFAP2A, which is transcriptionally activated by TFAP2A. We further demonstrate that miR-8072-mediated downregulation of TFAP2A leads to decreased *SNAI1* expression, thereby exerting a suppressive effect on the EMT process in TNBC. These findings not only reinforce the pivotal role of the miR-8072/TFAP2A axis in the suppression of TNBC, but also highlight its potential as a promising therapeutic target for this aggressive subtype of breast cancer.

## Conclusion

Our study provides compelling evidence supporting the inhibitory role of miR-8072 in both the proliferation and migratory/invasive ability of TNBC. These effects are achieved by downregulating TFAP2A, which subsequently inhibits the transcription of *SNAI1*. Our findings not only unravel a previously unrecognized role and mechanism underlying the anti-tumorigenic properties of miR-8072 in TNBC, but also present the potential of miR-8072 as a promising molecular biomarker or therapeutic target for TNBC. Additionally, the discovery of TFAP2A as a transcription activator of *SNAI1* enhances our understanding of the complex molecular pathways involved in TNBC progression, emphasizing TFAP2A as a key regulator in this aggressive subtype of breast cancer.

### Supplementary Information

Additional file 1.

Additional file 2.

## Data Availability

All data generated or analyzed during this study are included in this published article and its supplementary information files.

## Abbreviations

TNBC: Triple-negative breast cancer
EMT: Epithelial-mesenchymal transition
TFAP2A: Transcription factor AP-2α
