# Supplementary figures and images for: TFAP2A downregulation mediates tumor-suppressive effect of miR-8072 in triple-negative breast cancer via inhibiting SNAI1 transcription
Source: Breast Cancer Res. 2024 Jun 18;26:103. doi: 10.1186/s13058-024-01858-x (PMC11186287; doi:10.1186/s13058-024-01858-x)

**Fig.2E**

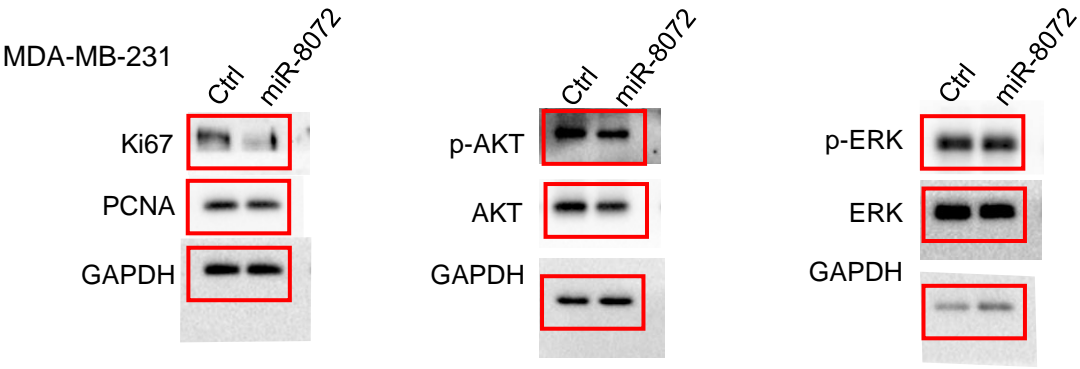

**Fig.2F**

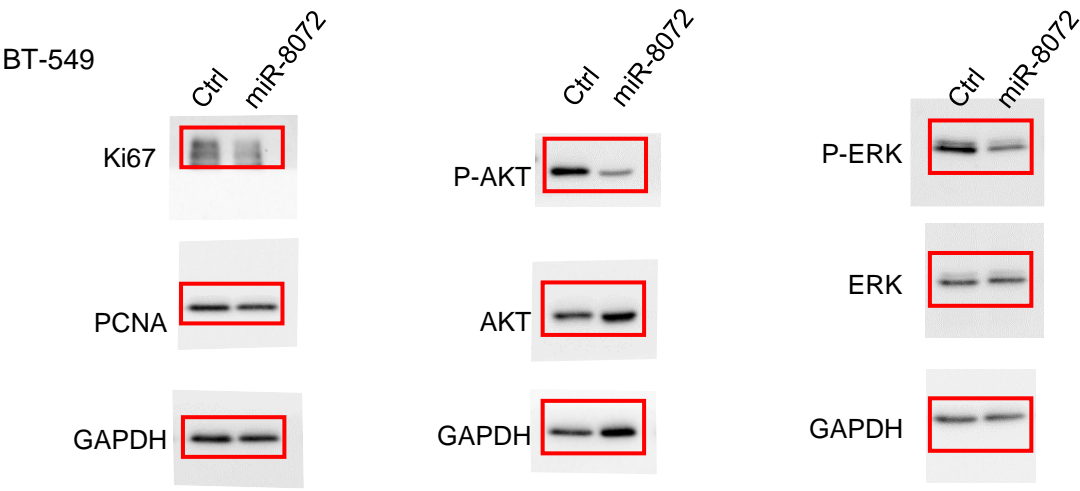

**Fig.3E**

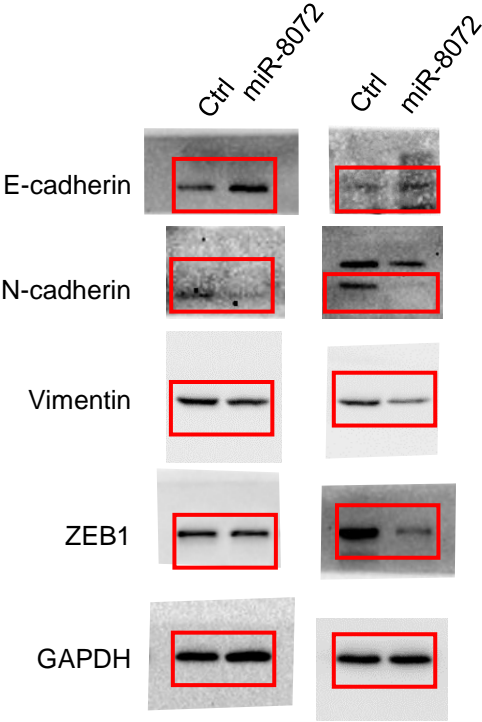

**Fig.4E**

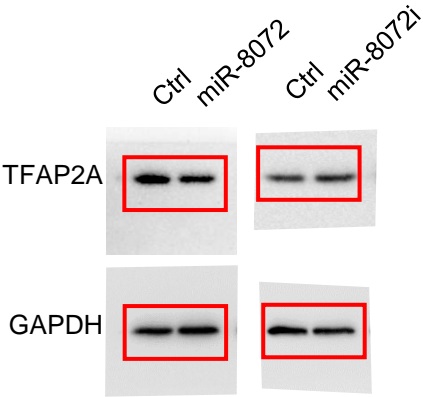

**Fig.5D**

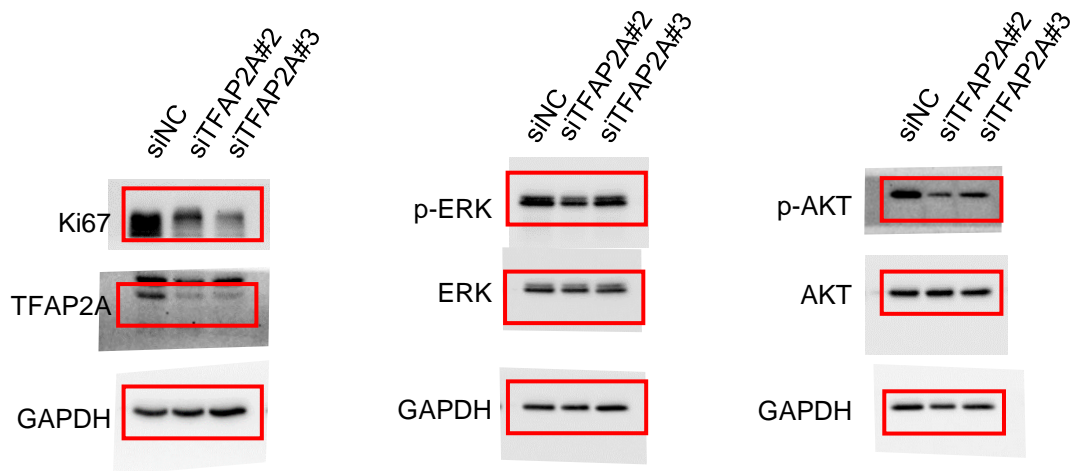

**Fig.5E**

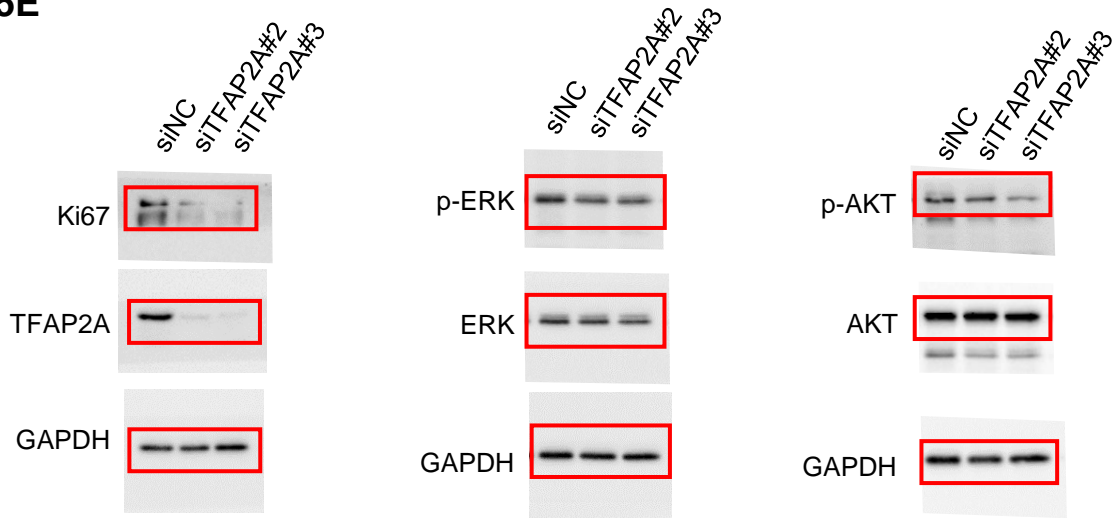

**Fig.6E**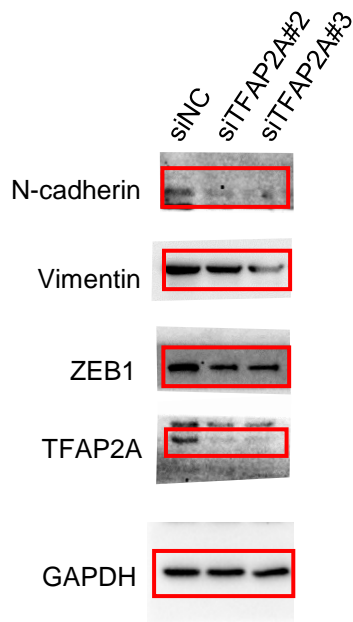**Fig.6F**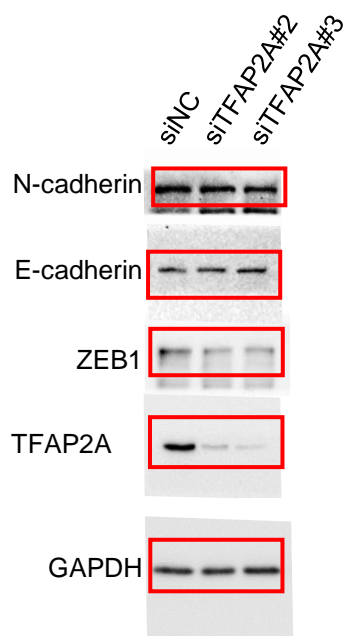**Fig.8E**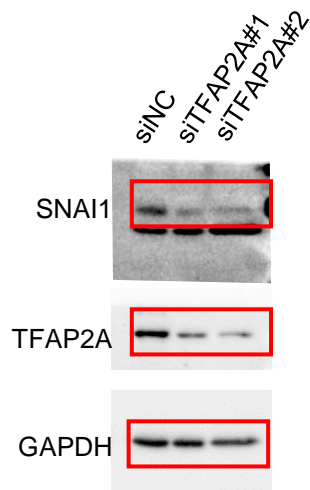**Fig.8F**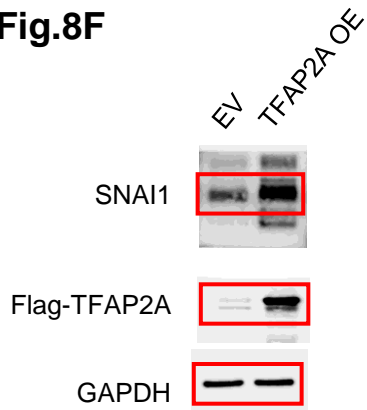**Fig.8G**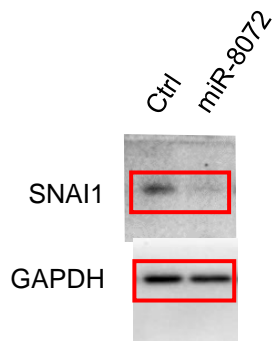**Fig.8H**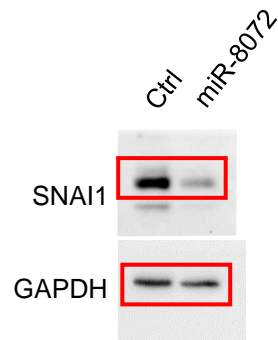

**Fig.S1D**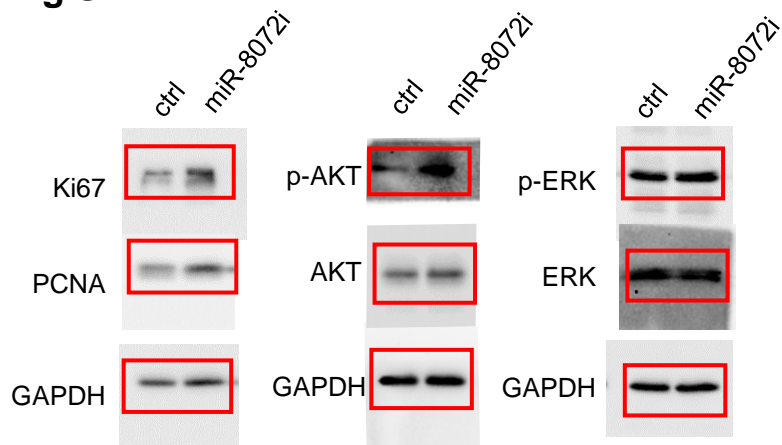**Fig.S2D**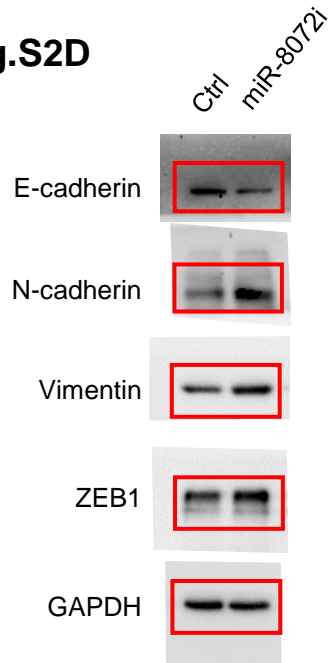**Fig.S3A**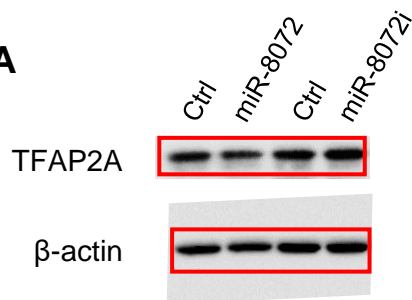**Fig.S4**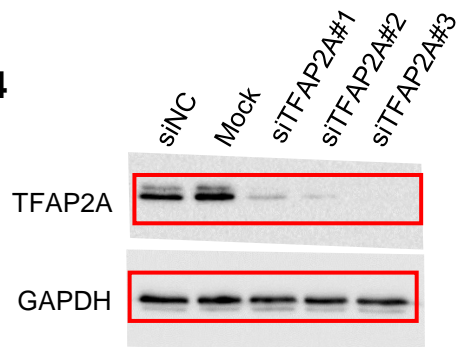**Fig.S6B**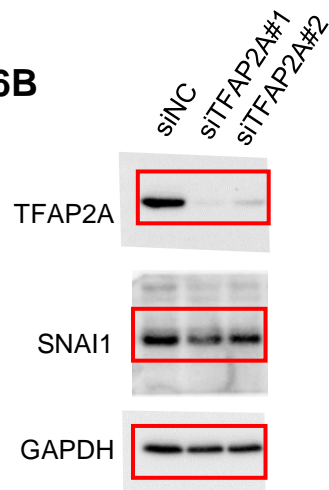

Supplement: Supplementary file 1 — Additional file 1. [file 13058_2024_1858_MOESM1_ESM.pdf]
