# Supplementary material for: TFAP2A downregulation mediates tumor-suppressive effect of miR-8072 in triple-negative breast cancer via inhibiting SNAI1 transcription
Source: Breast Cancer Res. 2024 Jun 18;26:103. doi: 10.1186/s13058-024-01858-x (PMC11186287; doi:10.1186/s13058-024-01858-x)
Supplement: Supplementary file 2 — Additional file 2. [file 13058_2024_1858_MOESM2_ESM.docx]

**Supplementary Figures**


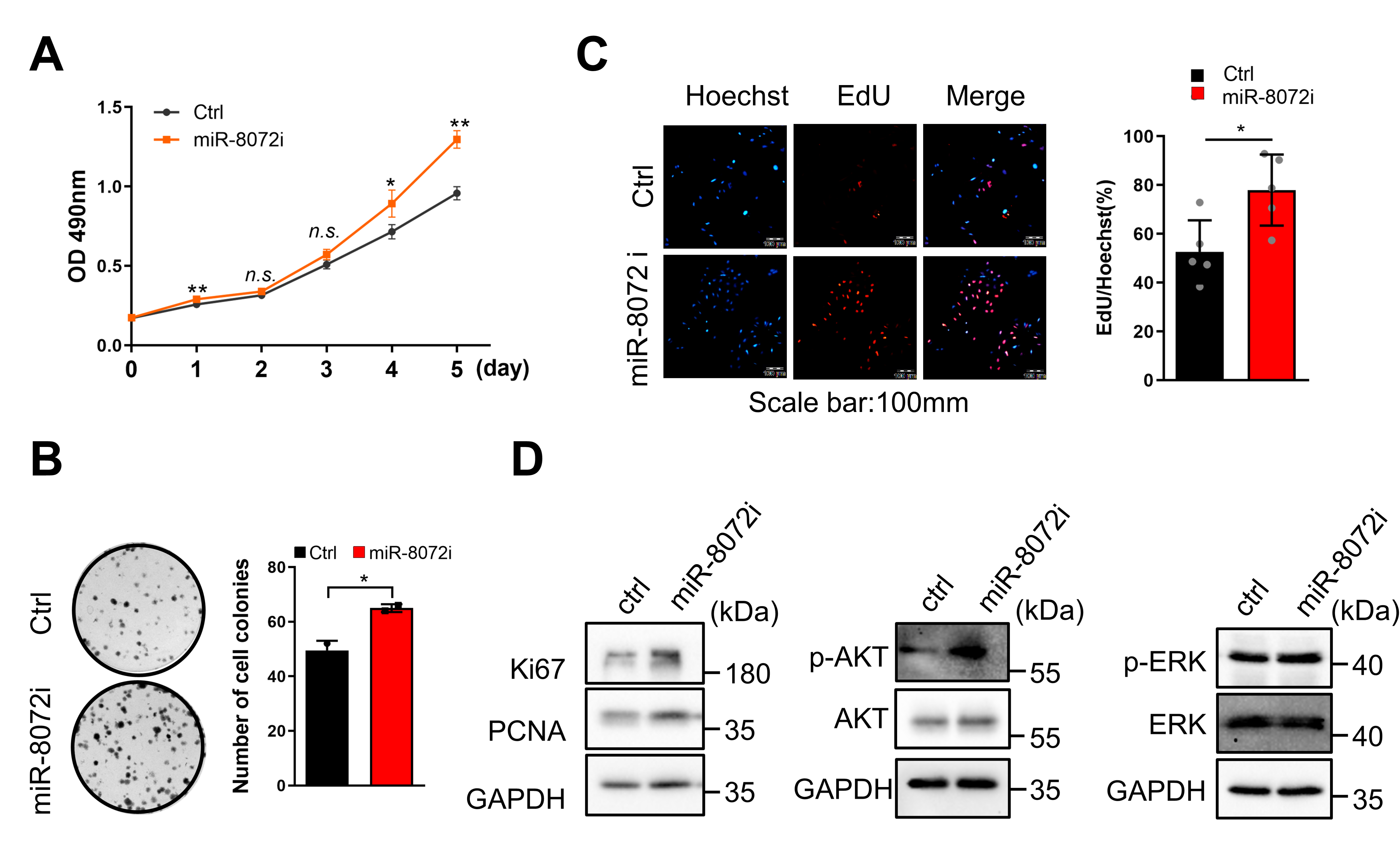


**Supplementary Figure 1.** Cell proliferation was measured by **(A)** MTS assay, **(B)** colony formation assay, and **(C)** EdU assay in MDA-MB-231 cells after functional inhibition of miR-8072. **(D)** Western blot analysis depicting the expression levels of proliferation-related protein levels or activation status in MDA-MB-231 cells after functional inhibition of miR-8072.


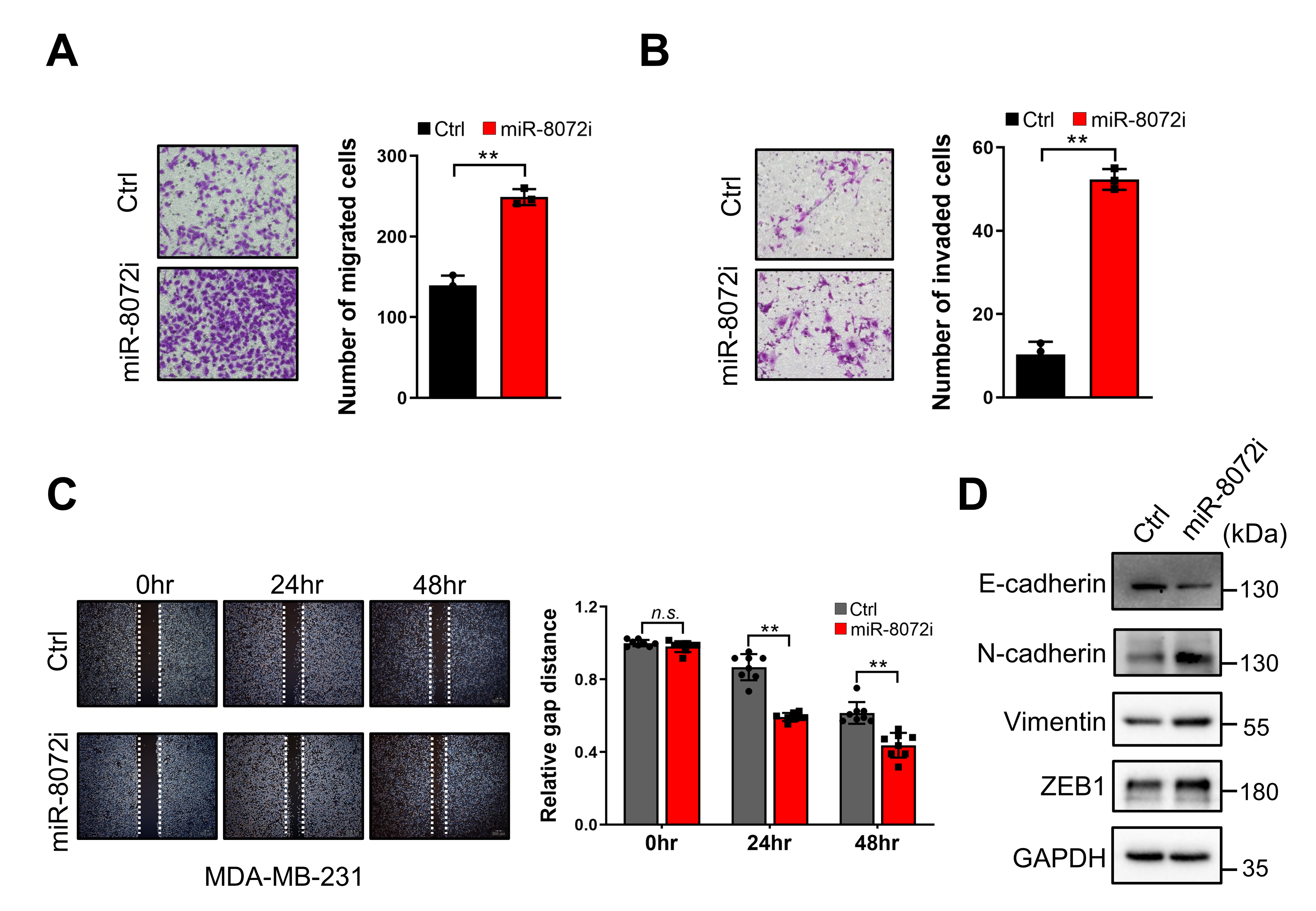


**Supplementary Figure 2.** Transwell assay demonstrating the **(A)** migration and **(B)** invasion abilities of MDA-MB-231 cells transduced with miR-8072 inhibitors or control. Representative images of migrated and invaded cells through the porous membrane are shown. **(C)** Scratch wound healing assay showing the migratory capacity of MDA-MB-231 cells transduced with miR-8072 inhibitors or control. Representative images were captured at 0, 24 and 48 hours post-scratch. **(D)** Western blot analysis depicting the expression levels of E-cadherin, N-cadherin, Vimentin, and ZEB1 proteins in MDA-MB-231 cells transduced with miR-8072 inhibitors or control.


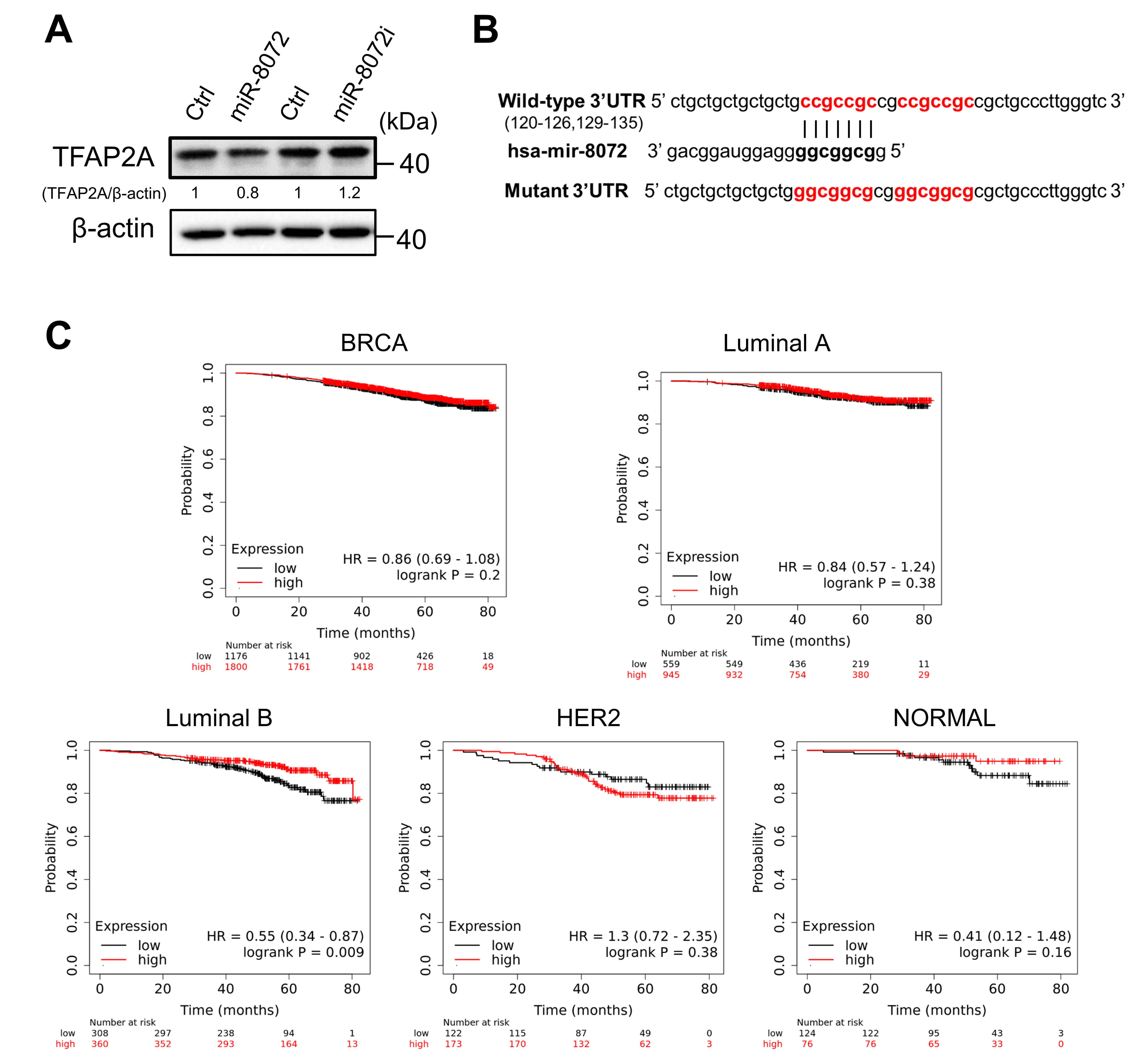


**Supplementary Figure 3.** ***TFAP2A* is a target gene of miR-8072 and is associated with unfavorable prognosis in TNBC. (A)** Evaluation of TFAP2A protein levels upon modulation of miR-8072 expression in MDA-MB-231 cells. **(B)** Schematic graph of the putative binding sites of miR-8072 in the TFAP2A 3′UTR and the mutation in miR-8072 binding sites for luciferase reporter analysis. **(C)** Survival analysis assessing the impact of TFAP2A expression on overall survival in patients with breast cancer and its subtypes.


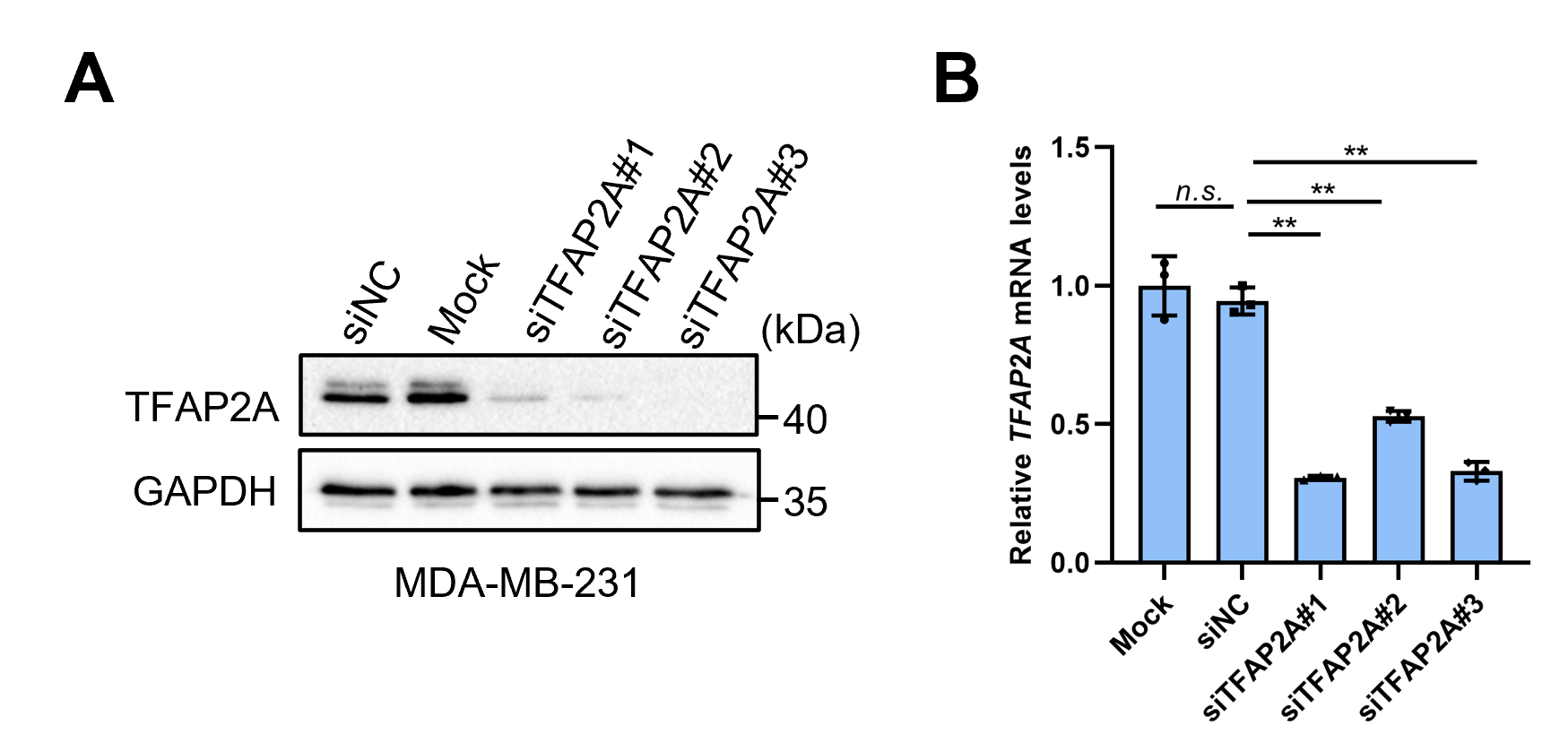


**Supplementary Figure 4. Evaluation of knockdown efficiency of TFAP2A by siRNAs.** MDA-MB-231 cells were transiently transfected with siRNAs targeting TFAP2A or scramble siRNA, 48 hours later, cells were collected for **(A)** western blot analysis and **(B)** qRT-PCR to test protein and mRNA levels of TFAP2A.


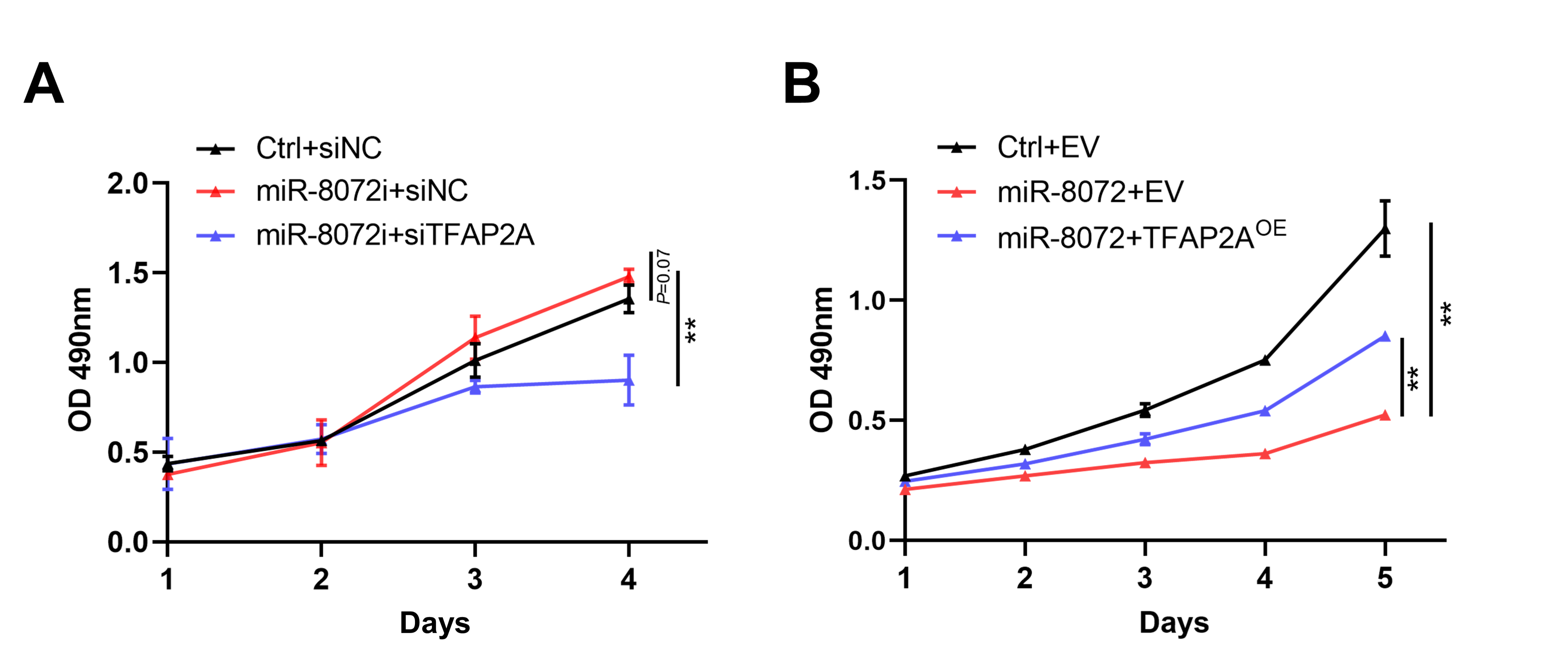


**Supplementary Figure 5. TFAP2A mediates tumor-suppressive effects of miR-8072 in TNBC cells. (A)** MDA-MB-231 cells were transfected with siTFAP2A or siNC in the presence or absence of miR-8072 functional inhibition; subsequently, MTS assay was performed to assess cell proliferation ability. (B) The MTS assay demonstrates that the overexpression of TFAP2A in miR-8072-overexpressing BT-549 cells reverses the inhibitory effects of miR-8072 on cell proliferation.

**
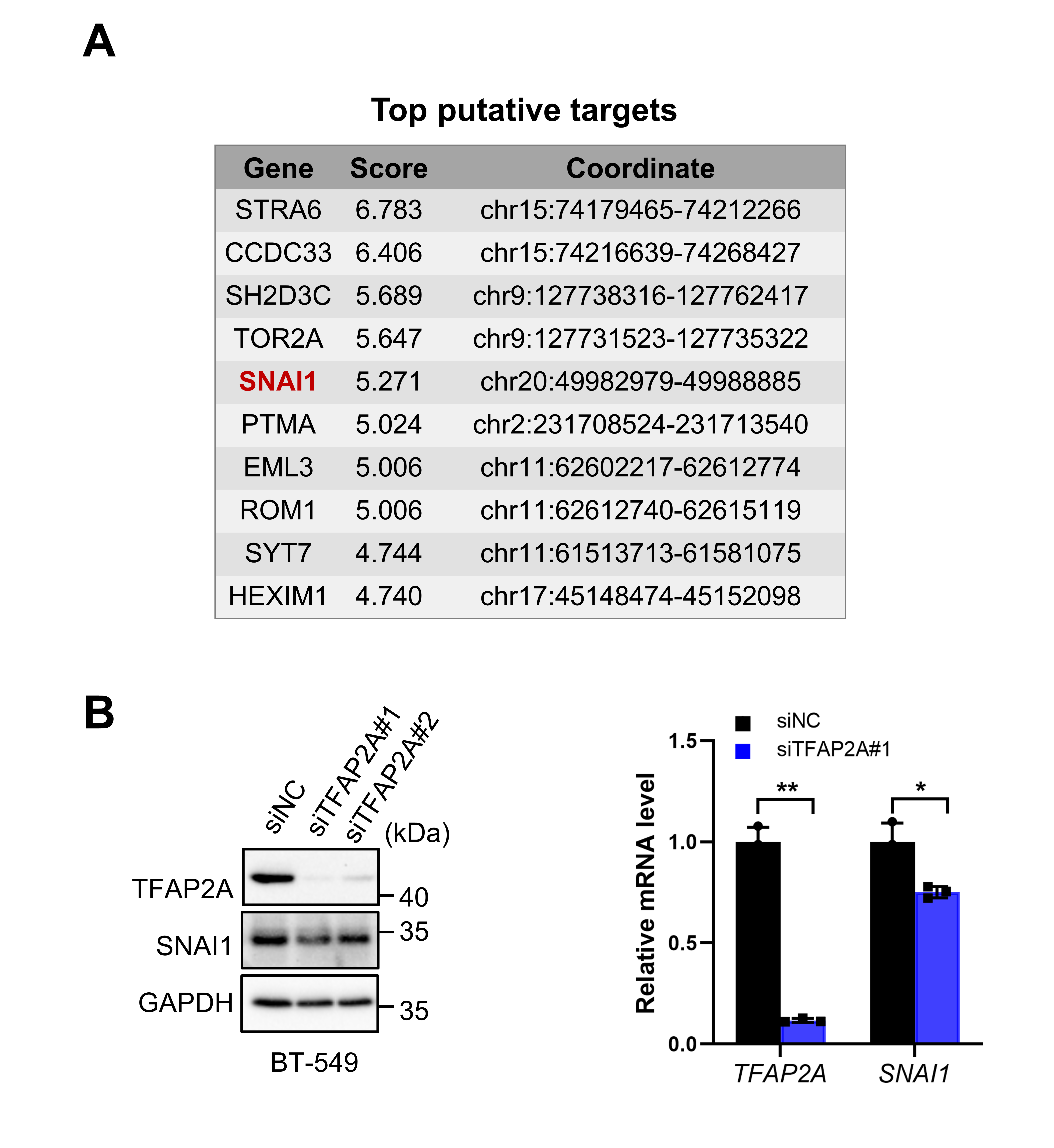
**

**Supplementary Figure 6. *SNAI1* is transcriptionally activated by TFAP2A. (A)** Top ten putative targets of TFAP2A in a public ChIP-seq data (http://dc2.cistrome.org, CistromeDB: 36958). **(B)** Protein and mRNA levels of SNAI1 were measured by western blot analysis and qRT-PCR, respectively, after silencing TFAP2A in BT-549 cells.
